# Supplementary material for: The fungal gene cluster for biosynthesis of the antibacterial agent viriditoxin
Source: Fungal Biol Biotechnol. 2019 Jul 1;6:2. doi: 10.1186/s40694-019-0072-y (PMC6600887; doi:10.1186/s40694-019-0072-y)

Table S1. Genome sequencing statistics for the assembly of *Aspergillus viridinutans* strain FRR 0576. The assembly and raw reads are available from GenBank under BioProject PRJNA513223.

Number of contigs	285
Median length (bp)	5,707
Mean length (bp)	104,615
Max length (bp)	1,359,167
N50 length (bp)	425,947
Number of contigs >N50	21
Length sum (bp)	29,815,307

Table S2. Protein IDs and features of the *Vdt* cluster genes in *Paecilomyces variotii* strains CBS 101075 and CBS 144490.

Protein	Protein ID CBS 101075	Protein ID CBS 144490	Putative function	Similarity/query cover to homologs discussed in the main text
VdtA	480069	260870	Polyketide synthase	42/94% to PKS12 (*F. graminearum*; aurofusarin)
VdtB	480050	260889	Laccase	53/86% to GIP1 (*F. graminearum*; aurofusarin)
VdtC	488617	127223	*O*-methyltransferase	44/96% to AurJ (*F. graminearum*; aurofusarin)
VdtD	510289	190631	Hydrolase-like	N/A
VdtE	480056	190779	Baeyer-Villiger monooxygenase	26/87% to MoxY (*A. parasiticus*; aflatoxin)
VdtF	480057	260883	Reductase	29/91% to FabG (*E. coli*)
VdtG	488624	275282	Major facilitator superfamily transporter	49/89% to AurT (*F. graminearum*; aurofusarin)
VdtR	105452	288289	Transcription factor	39/37% to AurR1 (*F. graminearum*; aurofusarin)
VdtX	515060	190767	PKS-like	37/97% to Fum1 (*Fusarium verticillioides*)

Table S3. Structural information of 1 (chloroform-*d*).

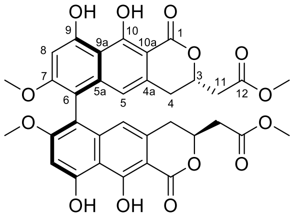

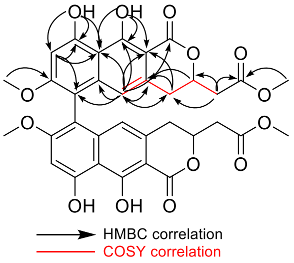

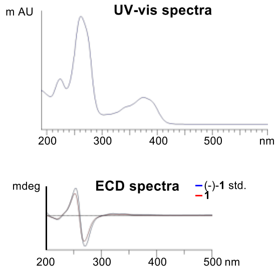

Carbon No.	^{13}C NMR	^{1}H NMR (ppm, multi, *J*)	gCOSY	HMBC
1	171.0	-	-	-
2	-	-	-	-
3	75.9	4.96 (1H, m)	4, 11	4a
4	33.0	2.81 (2H, m)	3, 5	4a, 5, 10a
4a	132.1	-		-
5	114.2	6.23 (1H, s)	4	1, 3, 4a, 5a, 6, 9a, 10, 10a
5a	139.3	-	-	-
6	110.1	-	-	-
7	161.2	-	-	-
7-OMe	56.3	3.77 (3H, s)	-	7
8	98.4	6.79 (1H, s)	-	6, 9, 9a
9	159.3	-	-	-
9-OH	-	9.69 (1H, s)	-	8, 9, 9a
9a	108.1	-	-	-
10	163.5	-	-	-
10-OH	-	13.75 (1H, s)	-	9a, 10, 10a
10a	98.8	-	-	-
11	39.5	2.65 (1H, dd, *J*=7.74, 19.5)<br>2.88 (1H, dd, *J*= 8.16, 19.4)	4	3, 4, 12
12	169.8	-	-	-
12-OMe	52.2	3.70 (3H, s)	-	12

Table S4. Structural information of 1′ (chloroform-*d*).

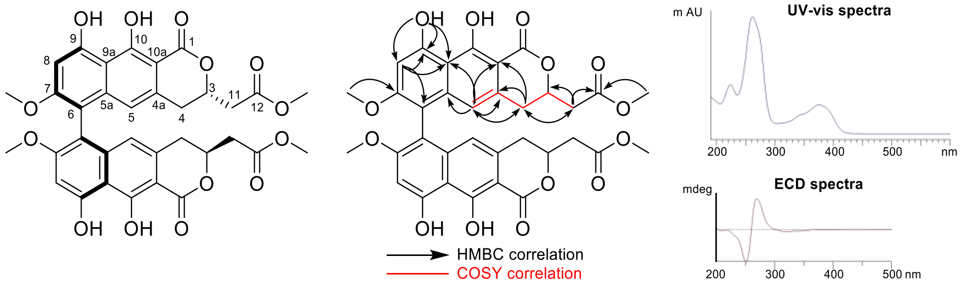

Carbon No.	^{13}C NMR	^{1}H NMR (ppm, multi, *J*)	gCOSY	HMBC
1	171.1	-	-	-
2	-	-	-	-
3	75.9	4.96 (1H, m)	4, 11	-
4	32.9	2.87 (2H, m)	3, 5	4a, 5, 10a, 11
4a	132.1	-		-
5	114.2	6.24 (1H, s)	4	4, 4a, 5a, 9a, 10a
5a	139.4	-	-	-
6	110.2	-	-	-
7	161.2	-	-	-
7-OMe	56.3	3.75 (3H, s)	-	7
8	98.3	6.79 (1H, s)	-	6, 9, 9a
9	159.3	-	-	-
9-OH	-	9.77 (1H, s)	-	8, 9, 9a
9a	108.2	-	-	-
10	163.6	-	-	-
10-OH	-	13.77 (1H, s)	-	-
10a	98.9	-	-	-
11	39.5	2.68 (1H, dd, *J*=6.55, 16.3)<br>2.92 (1H, dd, *J*= 6.70, 16.3)	4	3, 4, 12
12	169.9	-	-	-
12-OMe	52.3	3.70 (3H, s)	-	12

Table S5. Structural information of 2 (acetonitrile-d_3).

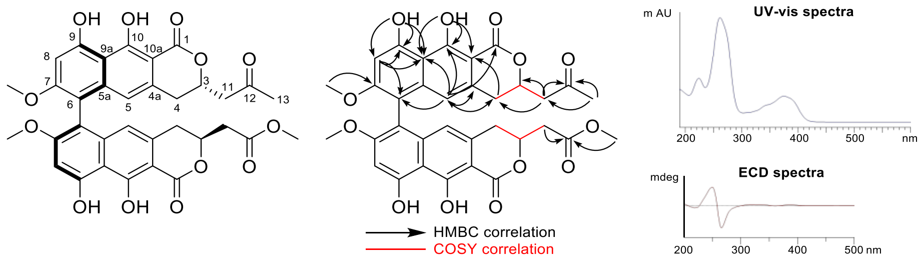

Carbon No.	^{13}C NMR	^{1}H NMR (ppm, multi, *J*)	gCOSY	HMBC
1	172.2	-	-	-
1′	172.0	-	-	
2 (2′)	-	-	-	-
3	76.8	4.96 (2H, m)	4, 11	-
3′	77.3	4.96 (2H, m)	4′, 11′	-
4	32.8	2.68[a]	3	5, 10a
4′	33.0	2.68[a]	3′	5′, 10a′
4a	134.2	-	-	-
4a′	134.0	-	-	-
5	114.8	6.28 (2H, s)	-	1, 4, 4a, 6, 9a, 10
5′	114.7	6.28 (2H, s)	-	1′, 4′, 4a′, 6′, 9a′, 10′
5a (5a′)	140.1	-	-	-
6 (6′)	110.9	-	-	-
7 (7′)	161.8	-	-	-
7 (7′)-OMe	56.8	3.71 (6H, s)	-	7 (7′)
8 (8′)	99.0	6.83 (1H, s)	-	6 (6′), 9 (9′), 9a (9a′)
9 (9′)	159.9	-	-	-
9 (9′)-OH	-	9.68 (2H, s)	-	8 (8′), 9 (9′), 9a (9a′)
9a (9a′)	108.5	-	-	-
10	163.7	-	-	-
10′	163.8	-	-	-
10 (10′)-OH	-	13.8 (1H, s)	-	9a (9a′), 10 (10′), 10a (10a′)
10a	100.0	-	-	-
10a′	99.9	-	-	-
11	48.3	2.74[a] 2.96 (1H, dd, *J*=7.65, 17.4)	3	3, 4, 12
11′	39.9	2.70[a]	3′	3′, 4′, 12′
12	205.7	-	-	-

12′	170.9	-	-	-
13	30.6	2.12 (3H, s)	-	11, 12
12′-OMe	52.4	3.65 (3H, s)	-	12′

[a] unable to integrate or calculate *J* due to signal overlay

Table S6. Structural information of 3 (acetonitrile-d_3).

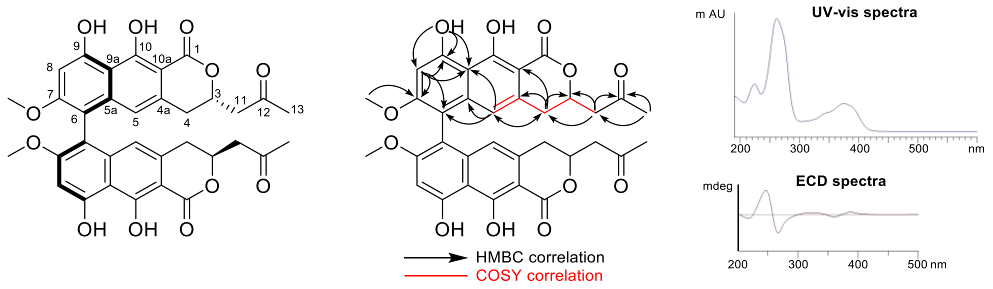

Carbon No.	^{13}C NMR	^{1}H NMR (ppm, multi, *J*)	gCOSY	HMBC
1	172.2	-	-	-
2	-	-	-	-
3	76.8	4.98 (1H, m)	4, 11	-
4	33.0	2.79 (2H, m)	3, 5	3, 4a, 5, 10a
4a	134.4	-		-
5	114.7	6.27 (1H, s)	4	4, 5a, 6, 9a
5a	140.2	-	-	-
6	110.9	-	-	-
7	161.8	-	-	-
7-OMe	56.8	3.71 (3H, s)	-	7
8	99.0	6.84 (1H, s)	-	6, 7, 9, 9a
9	159.9	-	-	-
9-OH	-	9.73 (1H, s)	-	8, 9, 9a
9a	108.6	-	-	-
10	163.8	-	-	-
10-OH	-	13.84 (1H, s)	-	-
10a	100.1	-	-	-
11	48.3	2.76 (1H, dd, *J*=7.92, 17.5)<br>2.96 (1H, dd, *J*= 7.74, 17.5)	4	3, 4, 12
12	205.7	-	-	-
13	30.6	2.11 (3H, s)	-	11, 12

Table S7. Structural information of 4 (acetonitrile-d_3).

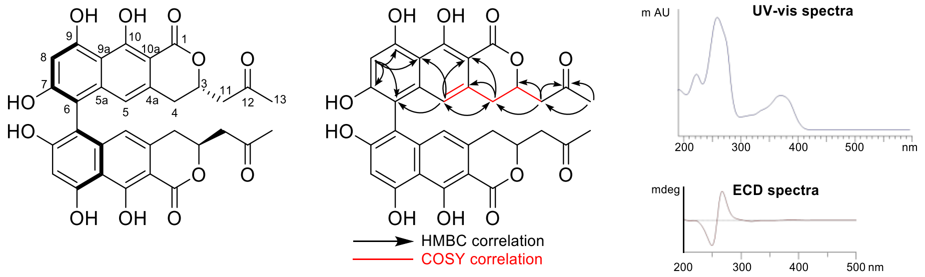

Carbon No.	^{13}C NMR	^{1}H NMR (ppm, multi, *J*)	gCOSY	HMBC
1	172.2	-	-	-
2	-	-	-	-
3	76.8	4.92 (1H, m)	4, 11	-
4	33.1	2.79[a]	3, 5	4a, 5, 10a
4a	135.0	-		-
5	114.4	6.35 (1H, s)	4	4, 9a, 10a
5a	141.5	-	-	-
6	109.2	-	-	-
7	160.5	-	-	-
7-OH	-	-	-	-
8	102.7	6.60 (1H, s)	-	6, 7, 9, 9a
9	160.0	-	-	-
9-OH	-	-	-	-
9a	107.0	-	-	-
10	163.6	-	-	-
10-OH	-	-	-	-
10a	100.3	-	-	-
11	48.3	2.78[a]<br>2.95 (1H, dd, *J*=7.45, 17.45)	3	3, 4, 12
12	205.9	-	-	-
13	30.6	2.11 (3H, s)	-	11, 12

[a] unable to integrate or calculate *J* due to signal overlay

Table S8. Structural information of 5 (acetonitrile-d_3).

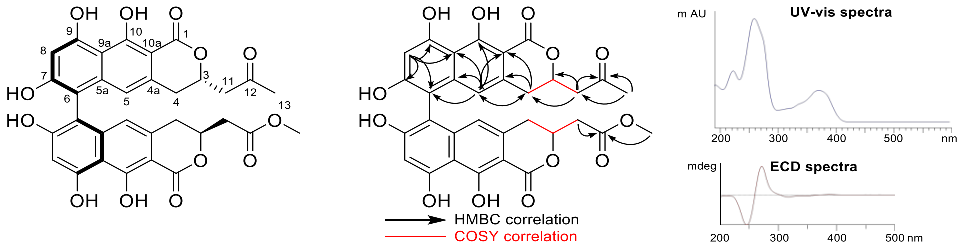

Carbon No.	^{13}C NMR	^{1}H NMR (ppm, multi, *J*)	gCOSY	HMBC
1	172.2	-	-	-
1′	172.0	-	-	
2 (2′)	-	-	-	-
3	76.8	4.89 (1H, m)	4, 11	-
3′	77.3	4.89 (1H, m)	4′, 11′	-
4	33.0	2.81[a]	3	4a, 5, 10a
4′	32.8	2.81[a]	3′	4a′, 5′, 10a′
4a	135.0	-	-	-
4a′	134.7	-	-	-
5	114.5	6.36 (1H, s)	-	4, 5a, 6, 9a, 10, 10a
5′	114.4	6.36 (1H, s)	-	4′, 5a′,6′, 9a′, 10′, 10a′
5a (5a′)	141.5	-	-	-
6 (6′)	106.9	-	-	-
7 (7′)	160.5	-	-	-
7 (7′)-OH	-	-	-	-
8 (8′)	102.7	6.60 (1H, s)	-	6 (6′), 7 (7′), 9 (9′), 9a (9a′)
9 (9′)	160.0	-	-	-
9 (9′)-OH	-	-	-	-
9a (9a′)	109.2	-	-	-
10	163.5	-	-	-
10′	163.6	-	-	-
10 (10′)-OH	-	-	-	-
10a	100.0	-	-	-
10a′	99.9	-	-	-
11	48.3	2.74[a]<br>2.96 (1H, dd, *J*=7.44, 17.52)	3	3, 4, 12
11′	39.9	2.70[a]	3′	3′, 4′, 12′
12	205.7	-	-	-

12′	170.0	-	-	-
13	30.6	2.10 (1.5H, s)	-	11, 12
12′-OMe	52.5	3.62 (1.5H, s)	-	12′

[a] unable to integrate or calculate *J* due to signal overlay

Table S9. Structural information of 7 (chloroform-*d*).

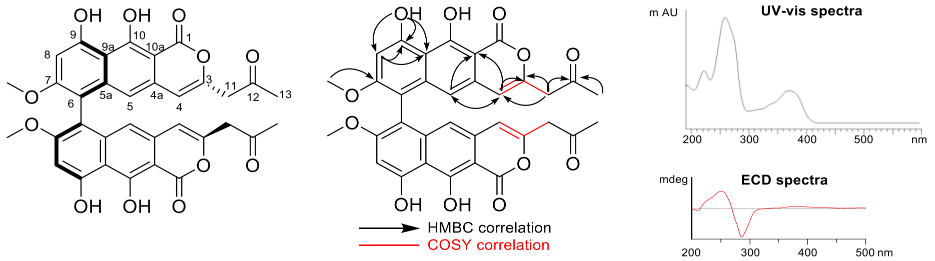

Carbon No.	^{13}C NMR	^{1}H NMR (ppm, multi, *J*)	gCOSY	HMBC
1	168.0[a]	-	-	-
2	-	-	-	-
3	148.4	-	-	-
4	108.3	6.10 (1H, s)]	11	3, 5, 10a
4a	-[b]	-		-
5	111.7	6.36 (1H, s)	-	4, 10a
5a	140.2[a]	-	-	-
6	109.9[a]	-	-	-
7	161.3	-	-	-
7-OMe	56.4	3.77 (3H, s)	-	7
8	98.4	6.84 (1H, s)	-	9, 9a
9	159.3	-	-	-
9-OH	-	9.75(1H, s)	-	8, 9, 9a
9a	-[b]	-	-	-
10	163.0[a]	-	-	-
10-OH	-	13.6 (1H, s)	-	-
10a	97.5	-	-	-
11	47.6	3.47 (2H, s)	4	3, 4, 12
12	202.2	-	-	-
13	30.1	2.23 (3H, s)	-	12

[a] Lacking HMBC correlations. Chemical shifts assigned based on other compounds isolated.
[b] ^{13}C signals not detected.

Table S10. Structural information of 8 (chloroform-*d*).

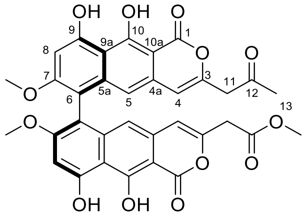

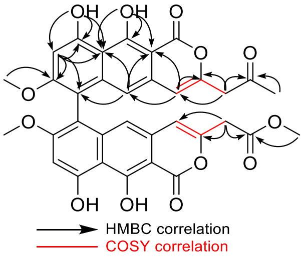

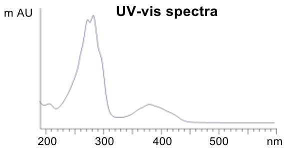

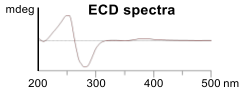

Carbon No.	^{13}C NMR	^{1}H NMR (ppm, multi, *J*)	gCOSY	HMBC
1	.168.0	-	-	-
1′	167.9	-	-	
2 (2′)	-	-	-	-
3	148.4	-	-	-
3′	147.9	-	-	-
4	108.3	6.10 (1H, s)	11	3, 5, 10a
4′	108.0	6.15 (1H, s)	11′	3′, 5′, 10a′
4a	130.2	-	-	-
4a′	130.5	-	-	-
5	111.7	6.36 (1 H, s)	-	6, 9a, 10a
5′	111.8	6.37 (1 H, s)	-	6′, 9a′, 10a′
5a (5a′)	140.2	-	-	-
6 (6′)	109.9	-	-	-
7 (7′)	161.3	-	-	-
7 (7′)-OMe	56.4	3.77 (6H, s)	-	7 (7′)
8 (8′)	98.4	6.84 (2H, s)	-	6 (6′), 7 (7′), 9 (9′), 9a (9a′)
9 (9′)	159.3	-	-	-
9 (9′)-OH	-	9.75 (2H, s)	-	8 (8′), 9 (9′), 9a (9a′)
9a (9a′)	108.2	-	-	-
10 (10′)	163.0	-	-	-
10 (10′)-OH	-	13.6 (2H, s)	-	9a (9a′), 10 (10a′), 9a (9a′)
10a	97.5	-	-	-
10a′	97.6	-	-	-
11	47.6	3.47 (2H, s)	4	3, 4, 12
11′	38.8	3.43 (2H, s)	4′	3′, 4′, 12′
12	202.2	-	-	-
12′	168.7	-	-	-

13	30.1	2.23 (3H, s)	-	12
12′-OMe	52.7	3.71 (3H, s)	-	12′

Table S11. Structural information of 9 (chloroform-*d*).

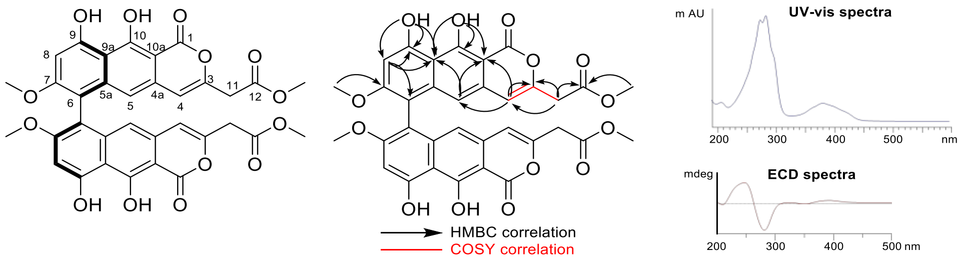

Carbon No.	^{13}C NMR	^{1}H NMR (ppm, multi, *J*)	gCOSY	HMBC
1	167.9[a]	-	-	-
2	-	-	-	-
3	147.9	-	-	-
4	108.0	6.15 (1H, s)	11	3, 5, 10a
4a	130.8[a]	-		-
5	111.8	6.37 (1H, s)	-	9a, 10a
5a	140.2[a]	-	-	-
6	110.0[a]	-	-	-
7	161.2	-	-	-
7-OMe	56.4	3.77 (3H, s)	-	7
8	98.5	6.84 (1H, s)	-	6, 9, 9a
9	159.3	-	-	-
9-OH	-	9.69	-	8, 9, 9a
9a	108.1	-	-	-
10	163.0	-	-	-
10-OH	-	13.75	-	9a, 10, 10a
10a	97.6	-	-	-
11	38.8	3.43 (2H, s)	4	3, 4, 12
12	168.7	-	-	-
12-OMe	52.7	3.71(3H, s)	-	12

[a] ^{13}C signals have no HMBC correlations. The chemical shifts were assigned based on data of other compounds.

Table S12. Oligonucleotide primers designed in this study

Name	Sequence (5′ to 3′)	Notes
vdtB5'F	AACAGCTATGACATGATTACGAATCGACGTTGTTTCTTGG	Primers used to amplify 5′ flank of *vdtB*
vdtB5'R	GTGGGCAGAATTCGCTCCATGGCAGCAATGTCC	
vdtC5'F	AACAGCTATGACATGATTACGTGCTTCTTTATACTGGGTCG	Primers used to amplify 5′ flank of *vdtC*
vdtC5'R	TTGAAGAGAATTCGAAGCACTGATTGCCTGTGC	
vdtD5'F	AACAGCTATGACATGATTACGCCTTCTTCAAGTATCTTTCC	Primers used to amplify 5′ flank of *vdtD*
vdtD5'R	ACATACGGAATTCTGGTAACGGAAGGAAAAGACC	
vdtE5'F	AACAGCTATGACATGATTACGGGTTTGTAGGGATCGAATGC	Primers used to amplify 5′ flank of *vdtE*
vdtE5'R	CCGAAGGGAATTCCAACTCTGAAATACATCTCC	
vdtF5'F	AACAGCTATGACATGATTACGCGTCAATCGAGTTGAAATGG	Primers used to amplify 5′ flank of *vdtF*
vdtF5'R	CAGTCGAGAATTCGTCTGTTCTGTTGTTGGTGC	
vdtG5'F	AACAGCTATGACATGATTACGGGTCTGTTCTGTTGTTGGTGC	Primers used to amplify 5′ flank of *vdtG*
vdtG5'R	CCTCAGGGAATTCTGATCGAGCGCCAACCTACC	
vdtA5'F	AACAGCTATGACATGATTACGTTCTTATATACTTATAATTCG	Primers used to amplify 5′ flank of *vdtA*
vdtA5'R	GAGAACGGGATCCATAAACGACGCAAGCTGG	
vdtR5'F	AACAGCTATGACATGATTACGGCGTGCGTAGTAGCTGCTGC	Primers used to amplify 5′ flank of *vdtR*
vdtR5'R	AGATTGGAATTCTTGAGATAGGGTTTGACTGG	
vdtB3'F	ATGGAGCGAATTCTGCCCACGGAGACGATATGC	Primers used to amplify 3′ flank of *vdtB*
vdtB3'R	GTAAAACGACGGCCAGTGCCAGGGCACACAAAGAATCTAGG	
vdtC3'F	GTGCTTCGAATTCTCTTCAAGTATCTTTCCAGC	Primers used to amplify 3′ flank of *vdtC*
vdtC3'R	GTAAAACGACGGCCAGTGCCAGGTAACGGAAGGAAAAGACC	
vdtD3'F	GTTACCAGAATTCCGTATGTCTTTAATGAGTTGG	Primers used to amplify 3′ flank of *vdtD*
vdtD3'R	GTAAAACGACGGCCAGTGCCAATAATAATGTATCCGCTAGG	
vdtE3'F	AGAGTTGGAATTCCCTTCGGAGGAAAACGTACC	Primers used to amplify 3′ flank of *vdtE*
vdtE3'R	GTAAAACGACGGCCAGTGCCAATGCTAATATTCTGCCCTGC	
vdtF3'F	AACAGACGAATTCTCGACTGCCTGCTTCACTGG	Primers used to amplify 3′ flank of *vdtF*
vdtF3'R	GTAAAACGACGGCCAGTGCCAGGCTTCGACACAAACACTGG	
vdtG3'F	TCGATCAGAATTCCCTGAGGGGAGGAAACAAGG	Primers used to amplify 3′ flank of *vdtG*
vdtG3'R	GTAAAACGACGGCCAGTGCCAGCCCAGCCCATTACTGCAGC	
vdtA3'F	CGTTTATGGATCCCGTTCTCATTCGGTACAGC	Primers used to amplify 3′ flank of *vdtA*
vdtA3'R	GTAAAACGACGGCCAGTGCCAAAAAGGGGTGGATAATAA	

	GC	
vdtR3'F	TATCTCAAGAATTCCAATCTGCCCTCGAGTACG	Primers used to amplify 3′ flank of *vdtR*
vdtR3'R	GTAAAACGACGGCCAGTGCCAGTGCATTTCTTATACTCAAG G	
*vdtB*HYGF	GGACATTGCTGCCATGGAGCGCTGGGATTGCCCCTCGATGC	Primers used to amplify HYG cassette for introduction into the *vdtB* construct
*vdtB*HYGR	GCATATCGTCTCCGTGGGCAGCCTACTGAACGTTATGAC	
*vdtC*HYGF	CACAGGCAATCAGTGCTTCGCTGGGATTGCCCCTCGATGC	Primers used to amplify HYG cassette for introduction into the *vdtC* construct
*vdtC*HYGR	CTGGAAAGATACTTGAAGAGCCTACTGAACGTTATGAC	
*vdtD*HYGF	TCTTTTCCTTCCGTTACCAGCTGGGATTGCCCCTCGATGC	Primers used to amplify HYG cassette for introduction into the *vdtD* construct
*vdtD*HYGR	AACTCATTAAAGACATACGGCCTACTGAACGTTATGAC	
*vdtE*HYGF	GAGATGTATTTCAGAGTTGGCTGGGATTGCCCCTCGATGC	Primers used to amplify HYG cassette for introduction into the *vdtE* construct
*vdtE*HYGR	GTACGTTTTCCTCCGAAGGGCCTACTGAACGTTATGAC	
*vdtF*HYGF	CACCAACAACAGAACAGACGCTGGGATTGCCCCTCGATGC	Primers used to amplify HYG cassette for introduction into the *vdtF* construct
*vdtF*HYGR	CAGTGAAGCAGGCAGTCGAGCCTACTGAACGTTATGAC	
*vdtG*HYGF	GTAGGTTGGCGCTCGATCAGCTGGGATTGCCCCTCGATGC	Primers used to amplify HYG cassette for introduction into the *vdtG* construct
*vdtG*HYGR	CTTGTTTCCTCCCCTCAGGGCCTACTGAACGTTATGAC	
*vdtA*HYGF	CCAGCTTGCGTCGTTTATGCTGGGATTGCCCCTCGATGC	Primers used to amplify HYG cassette for introduction into the *vdtA* construct
*vdtA*HYGR	GCTGTACCGAATGAGAACGGCCTACTGAACGTTATGAC	
*vdtR*HYGF	CCAGTCAAACCCTATCTCAAGCTGGGATTGCCCCTCGATGC	Primers used to amplify HYG cassette for introduction into the *vdtR* construct
*vdtR*HYGR	CGTACTCGAGGGCAGATTGCCTACTGAACGTTATGAC	
AP124	AGTCGGCTGTTTGCATCTGC	488617ScF
AP125	GATCTGGGTCATAAACATCG	488617ScR
AP126	GAGGAGCAAGACAGAGACGG	510289scF
AP127	CTTGCATACTTGCCATTTTGG	510289scR
AP128	ATCATTGGGGAGATGGGAGG	480056ScF
AP129	GGTTATGTGTCGAATAAGCC	480056ScR
AP130	TGCCGTACCAACCGATATCG	480057scF
AP131	ATGTGATCATTCTAGCGACC	480057scR

AP132	CGGTTAATGCTTTTCCATCC	488624scF
AP133	TTTCTCTGATAGGACGATCC	488624scR
AP134	AATTGAGAGATTAGATAGCG	480069scF
AP135	GCAGATGGAGGAGCCATAGC	480069scR
AP136	TGGATGTCTTCCGGGTTGCG	105452_ScF
AP137	TTATTATGTGACACGTCTCG	105452_ScR
AP138	CAGCTTGATTTCCTCTGCC	480050ScF
AP139	TATAGGGGGGACCTTCAACC	480050ScR

qPCR primers

Name	Sequence (5´ to 3´)	Notes
vdtBqPCRF	CTATCACAGACAAAGGGTGC	qPCR primers for *vdtB*
vdtBqPCRR	AGGGAACACAGCCTTCCTGG	
vdtCqPCRF	GCGATCGTGGTTGATGTTGG	qPCR primers for *vdtC*
vdtCqPCRR	TCACCATGGGCAGGTCTTGC	
vdtDqPCRF	ACGGGTTCCGCGCATTTATGC	qPCR primers for *vdtD*
vdtDqPCRR	AGGATAGATATATTGGCTGG	
vdtEqPCRF	GAGCCGTTGAATTGGTTTGG	qPCR primers for *vdtE*
vdtEqPCRR	AAGACCGAAGAGAATATTCC	
vdtFqPCRF	CATCTACGGTGCGAGTAAGG	qPCR primers for *vdtF*
vdtFqPCRR	ATGTCGGTGTTGAAATATCC	
vdtGqPCRF	AGGATGCGGAATGAGCATGC	qPCR primers for *vdtG*
vdtGqPCRR	AAGAAGATGAGCGAGATACC	
vdtX1qPCRF	AGAGAGTAAACGTCTGTTGG	qPCR primers for *vdtX*
vdtXqPCRR	GCATTGTCGGATATCCGTCG	
vdtAqPCRF	AGCGACGGACAGATTTCTCG	qPCR primers for *vdtA*
vdtAqPCRR	TATAGGGTTCCTCCTGCAGC	
β-tubulinqPCRF	GGGCGAGGAGGAGTACAACG	qPCR primers for β-tubulin
β-tubulinqPCRR	AATGGGGTATTACTGAGAGC	
ID423248*qPCRF*	AGAGGATGGATTCAGAGAGC	qPCR primers for protein ID423248
ID423248*qPCRR*	GCAACGCGCGACGTTCTTCC	
AP169	TAAGGGCGATGATGTATTGG	qPCR primers for protein ID510298
AP170	GTCAGTAGGCGTCGATCTGC	
AP171	CCTGTGCTTTCTATAATGACC	qPCR primers for protein ID480071
AP172	CGTGATTCTGAAGGAGTGC	

Fluorescent tagging primers

Name	Sequence (5´ to 3´)
24GFP5F	AACAGCTATGACATGATTACGTATTCTGCTCTTCTTGAACC
24GFP5R	GCTCCTCGCCCTTGCTCACTACCTTGTTTCCTCCCCTCAGG
24GFPGFPF	GTGAGCAAGGGCGAGGAGC
24GFPGFPR	TAAAACGACGGCCAGTGCCAGAATTCGGTTGTTGGTGCTGGTGG
24GFPHYGF	CACCAGCACCAACAACCGCTGGGATTGCCCCTCGATGC
24GFPHYGR	GCTTACTATACATGCATATTTCCTACTGAACGTTATGAC

24GFP3F	AAATATGCATGTATAGTAAGC	
24GFP3R	AAAACGACGGCCAGTGCCAGGCCCAGCCCATTACTGCAGC	
69GFP5F	AACAGCTATGACATGATTACGAACAAGACGGCGACAAATCC	
69GFP5R	TCTAGCCGGATCCTAACTGCAAAACTCGAGAC	
69GFP3F	GCAGTTAGGATCCGGCTAGAGGAGCAGAGGTTGG	
69GFP3R	GTAAAACGACGGCCAGTGCCAGCAGATGGAGGAGCCATAGC	
69GFPHYGF	GTCTCGAGTTTTGCAGTTAGTGAGCAAGGGCGAGGAGCTG	
69GFPHYGR	AACCTCTGCTCCTCTAGCCGCCTACTGAACGTTATGAC	
Erg11F	TCGAAACCTAATCAATCAACATGGGGTTGCTCTCCGCTGTGC	
Erg11R	GCTCACCATTGCTTTTTCGGACAGTTGACG	
McherryErg11F	CCGAAAAAGCAATGGTGAGCAAGGGCGAGG	
McherryErg11R	TGCTCATAGTCACATCCCTCACTTGTACAGCTCGTCCATGC	
AP57	TCGAAACCTAATCAATCAACATGGTGAGCAAGGGCGAGG	
AP58	TGCTCATAGTCACATCCCTCAAAGCTTAGACTTGTACAGCTCGTCCATGC	
**Primers used for RIP mutation of *vdtX***		
**Oligo nucleotide**	**Sequence**	**Notes**
RIPF1	CCTCTGCAGGTCGACTCTAGACGATGCTAGATCACAAGTCC	Amplification and cloning of 2258 bp of *vdtX*
RIPR2	GGCCAATTCTTAATTAAGATATCGGCGAGAAATGATGTTTTCC	
RIPseqF	AATTTCCAACGATGCTCAGC	Amplification and sequencing of the native *vdtX* allele in the region targeted for RIP
RIPseqR	TTGGAACGAATCAGACAAGG	

Figure S1 UV-vis-spectra and mass spectra for 6, 10, 11, and 12

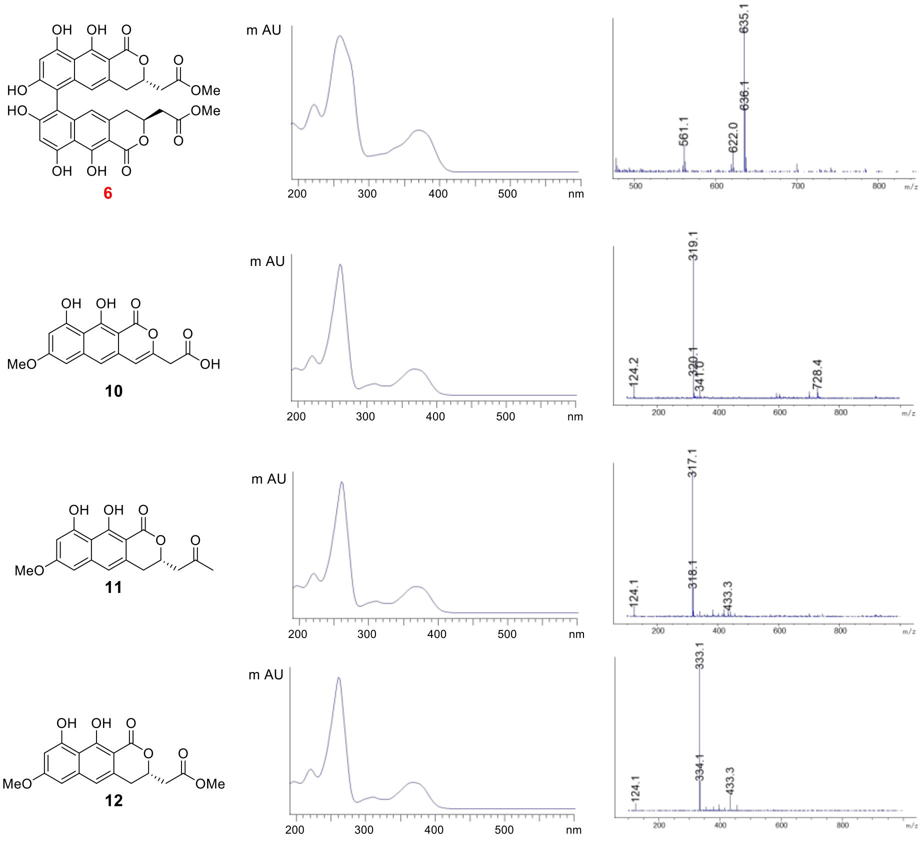

Figure S2. Mutation in *vdtX* via repeat induced point mutation. The top DNA and predicted amino acid sequences are from wild type and the bottom are from the RIP mutant.

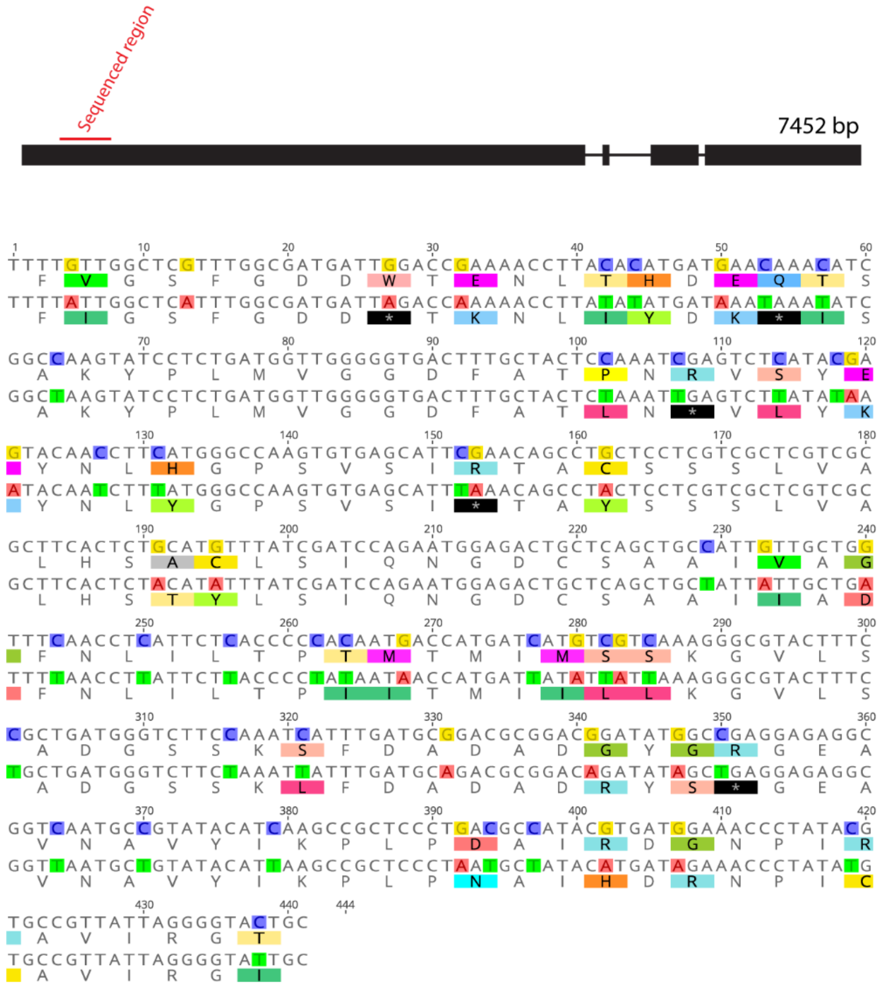

Figure S3. ^{1}H NMR spectrum (500 MHz) of 1 in chloroform-*d*

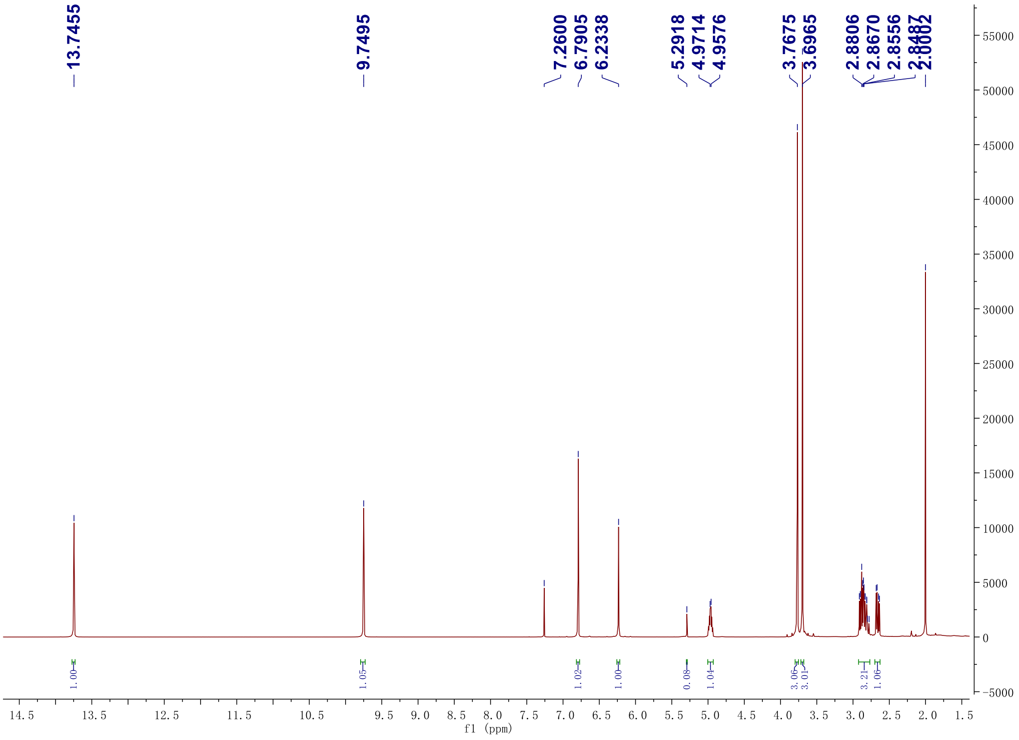

Figure S4. ^{13}C NMR spectrum (125 MHz) of 1 in chloroform-*d*

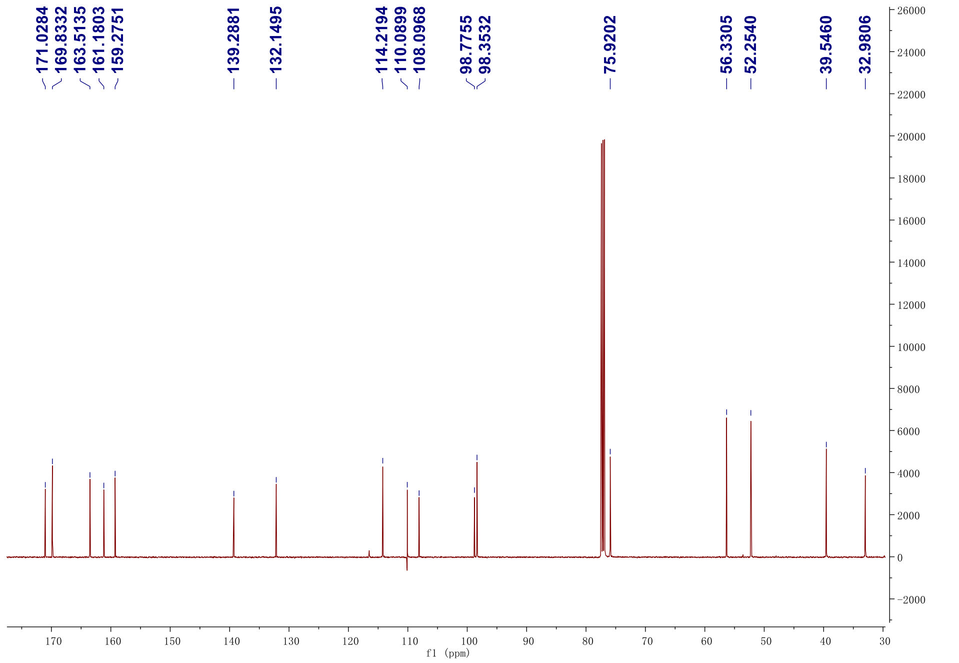

Figure S5. DEPT-135 ^{13}C NMR spectrum (125 MHz) of 1 in chloroform-*d*

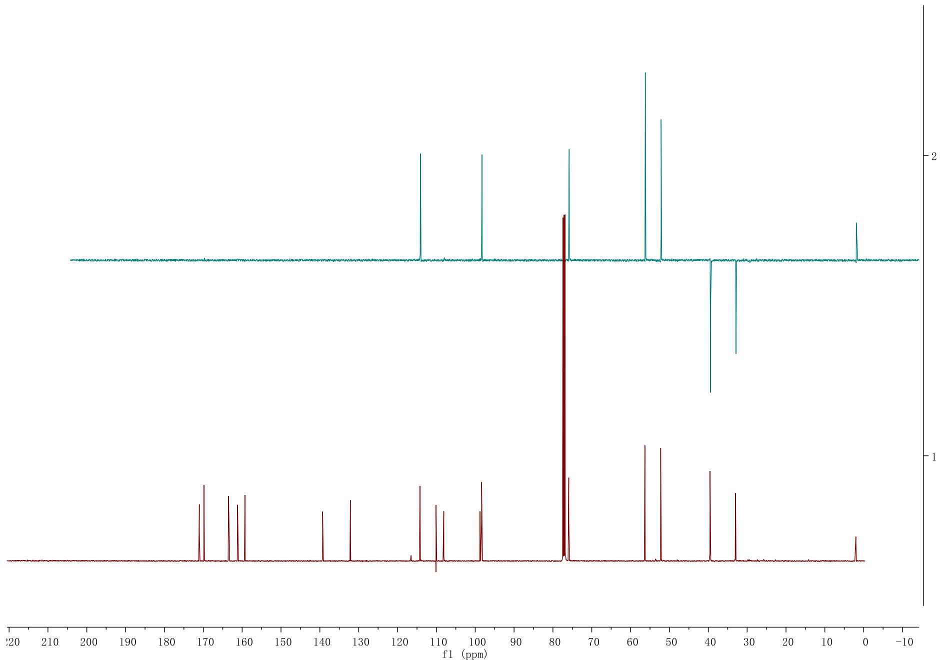

Figure S6. ^1H-^1H gCOSY NMR spectrum (500 MHz) of 1 in chloroform-*d*

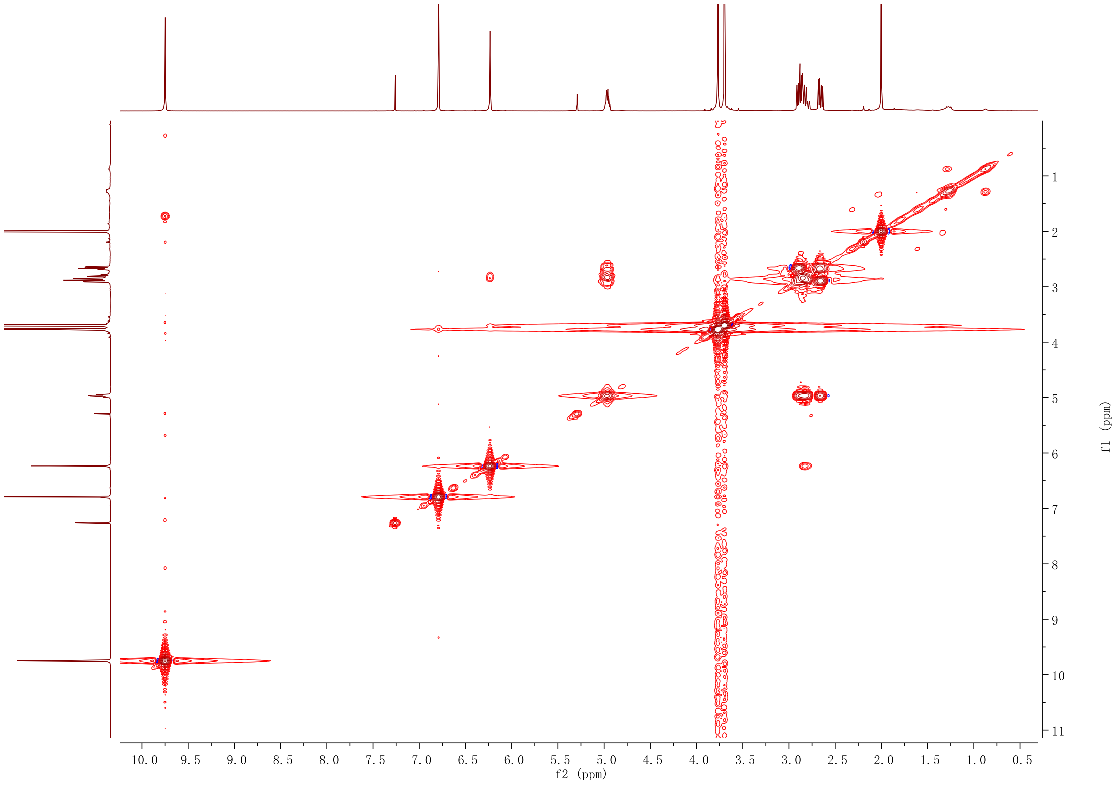

Figure S7. HSQC NMR spectrum (500 MHz) of 1 in chloroform-*d*

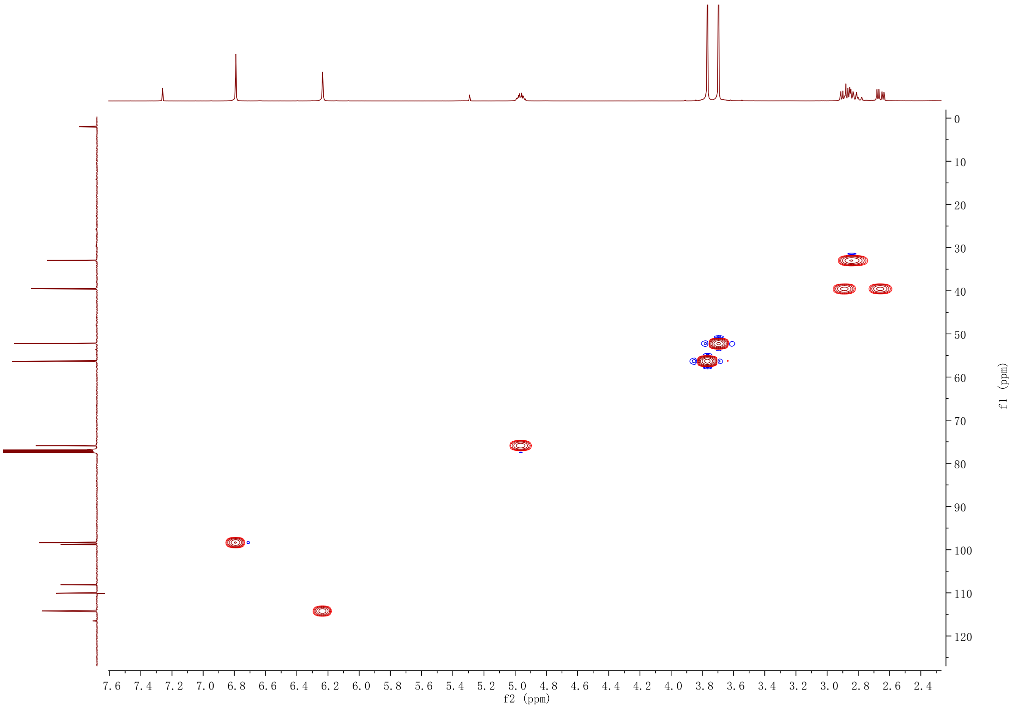

Figure S8. HMBC NMR spectrum (500 MHz) of 1 in chloroform-*d*

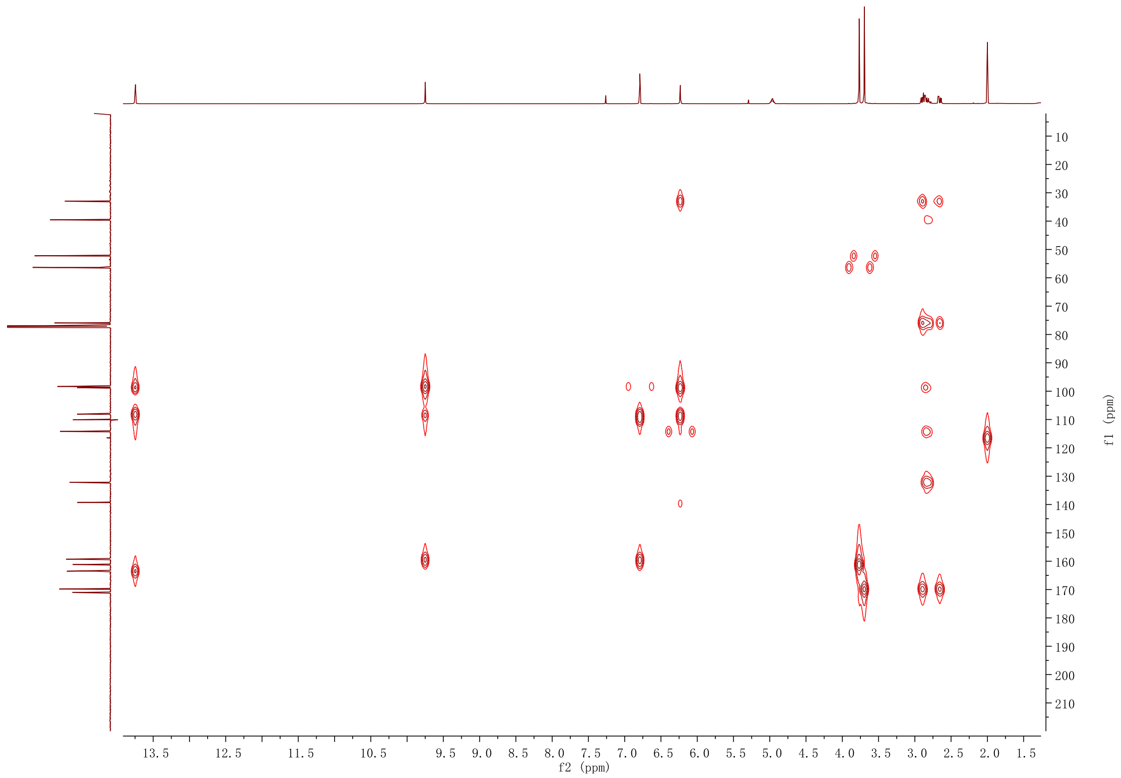

Figure S9. ^{1}H NMR spectrum (500 MHz) of 1′ in chloroform-*d*

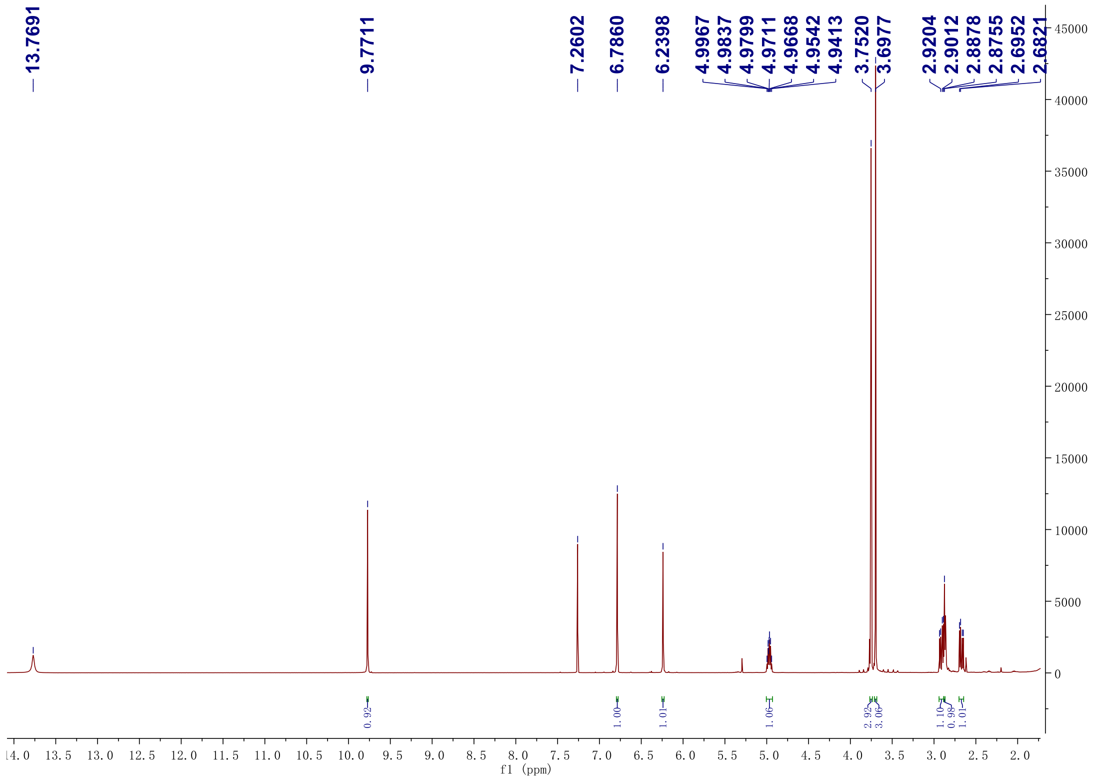

Figure S10. ^{13}C NMR spectrum (125 MHz) of 1′ in chloroform-*d*

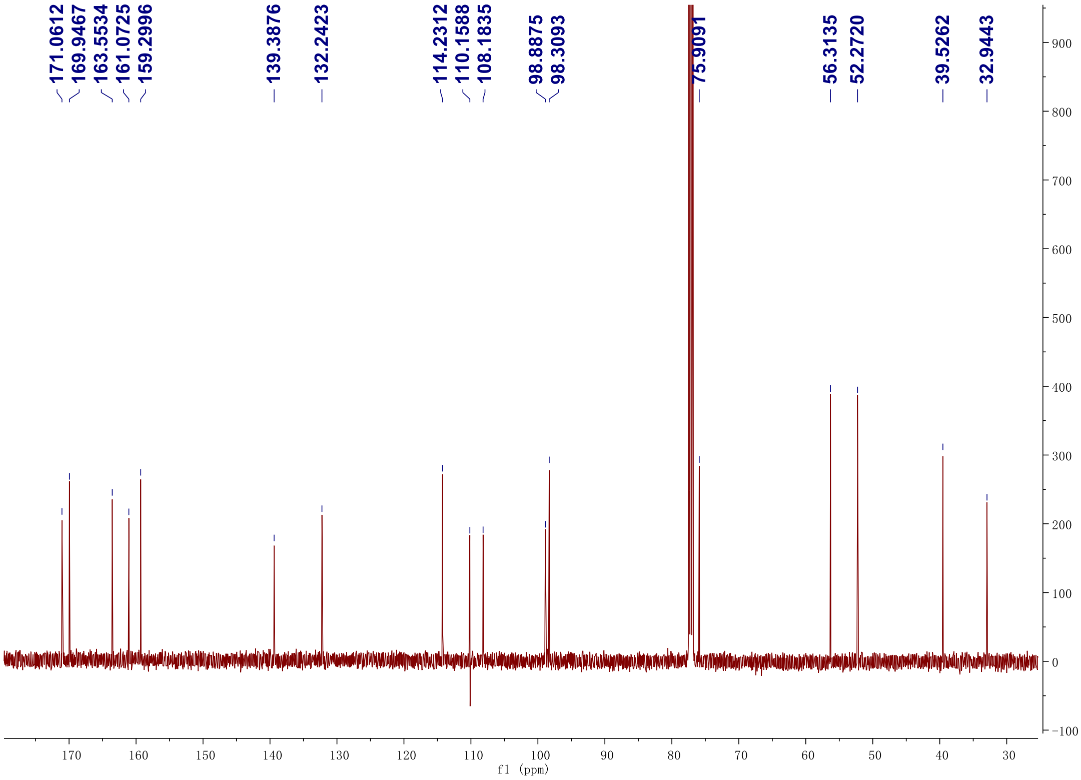

Figure S11. DEPT-135 ^{13}C NMR spectrum (125 MHz) of 1′ in chloroform-*d*

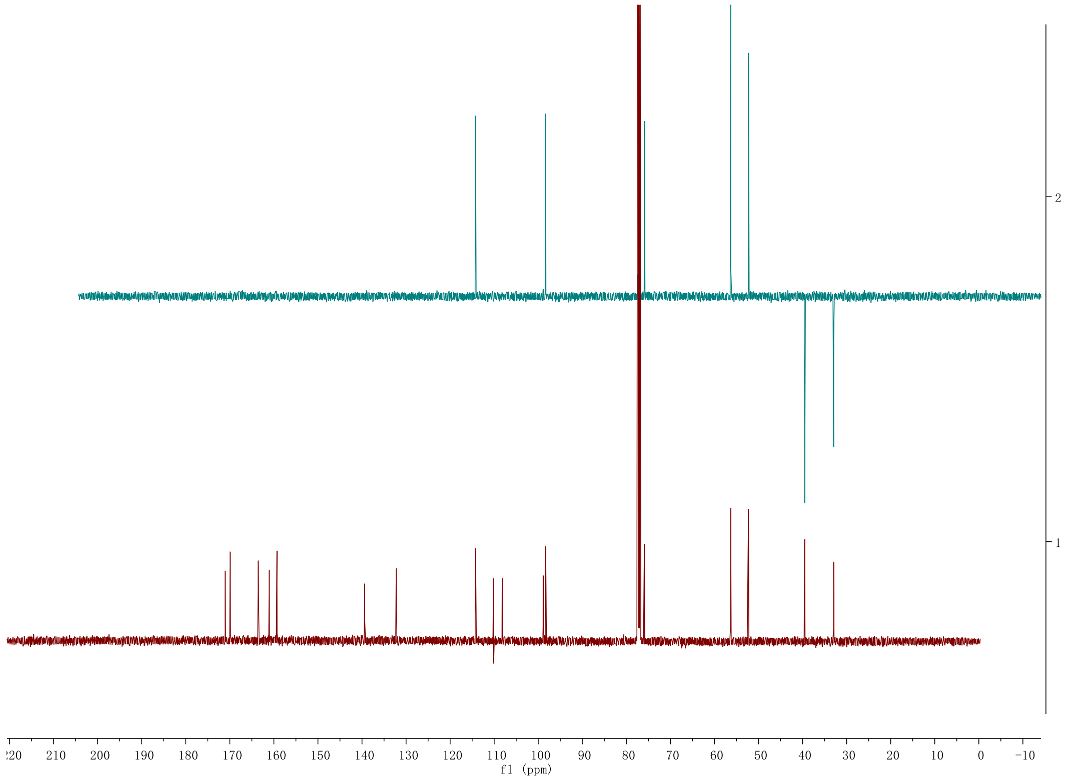

Figure S12. ^{1}H-^{1}H gCOSY NMR spectrum (500 MHz) of 1′ in chloroform-*d*

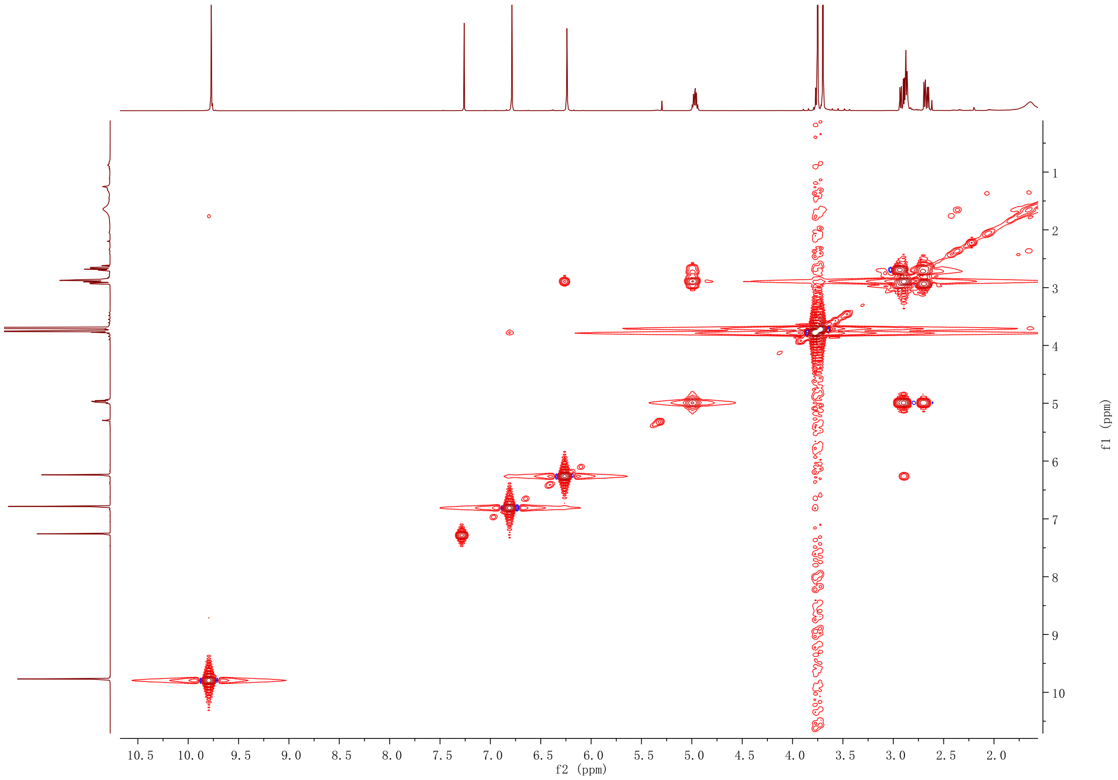

Figure S13. HSQC NMR spectrum (500 MHz) of 1′ in chloroform-*d*

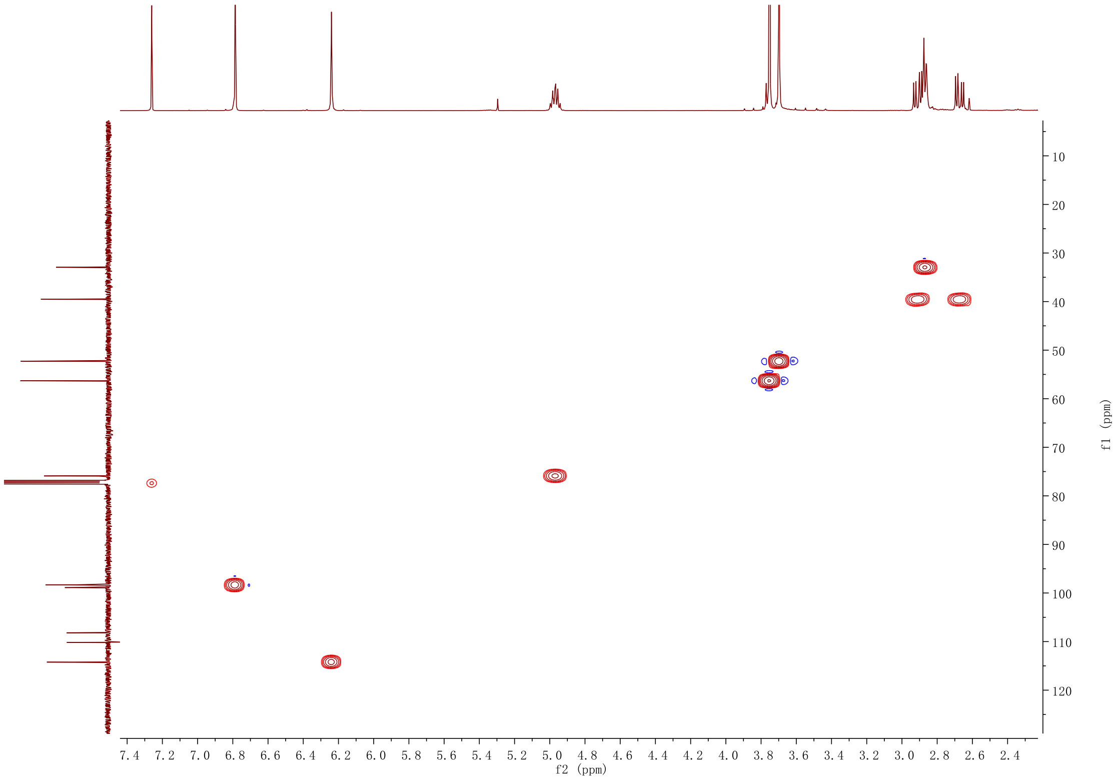

Figure S14. HMBC NMR spectrum (500 MHz) of 1′ in chloroform-*d*

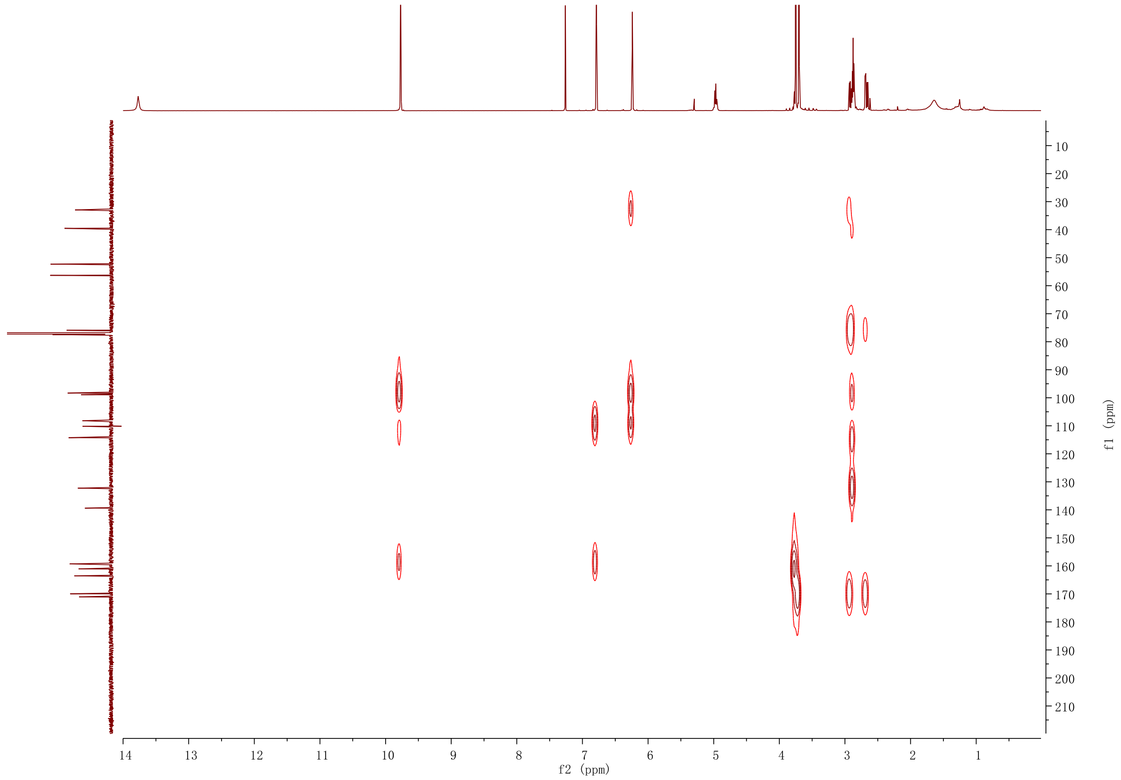

Figure S15. ^{1}H NMR spectrum (500 MHz) of 2 in acetonitrile-d_3

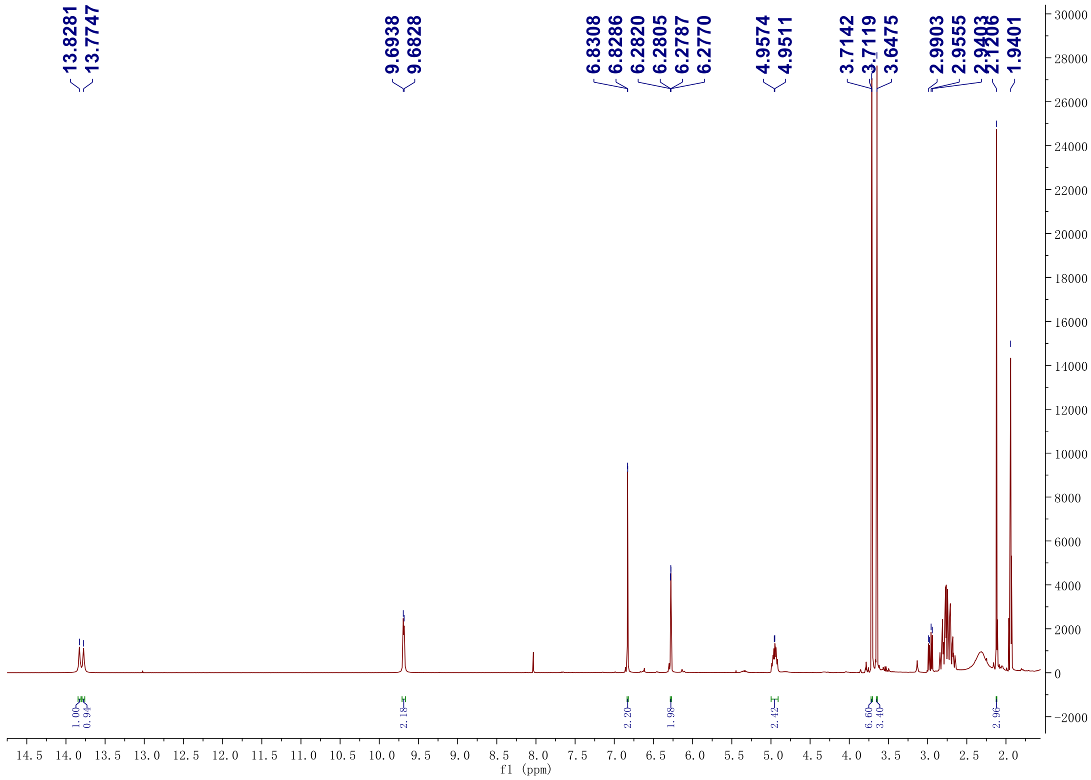

Figure S16. ^{13}C NMR spectrum (125 MHz) of 2 in acetonitrile-d_3

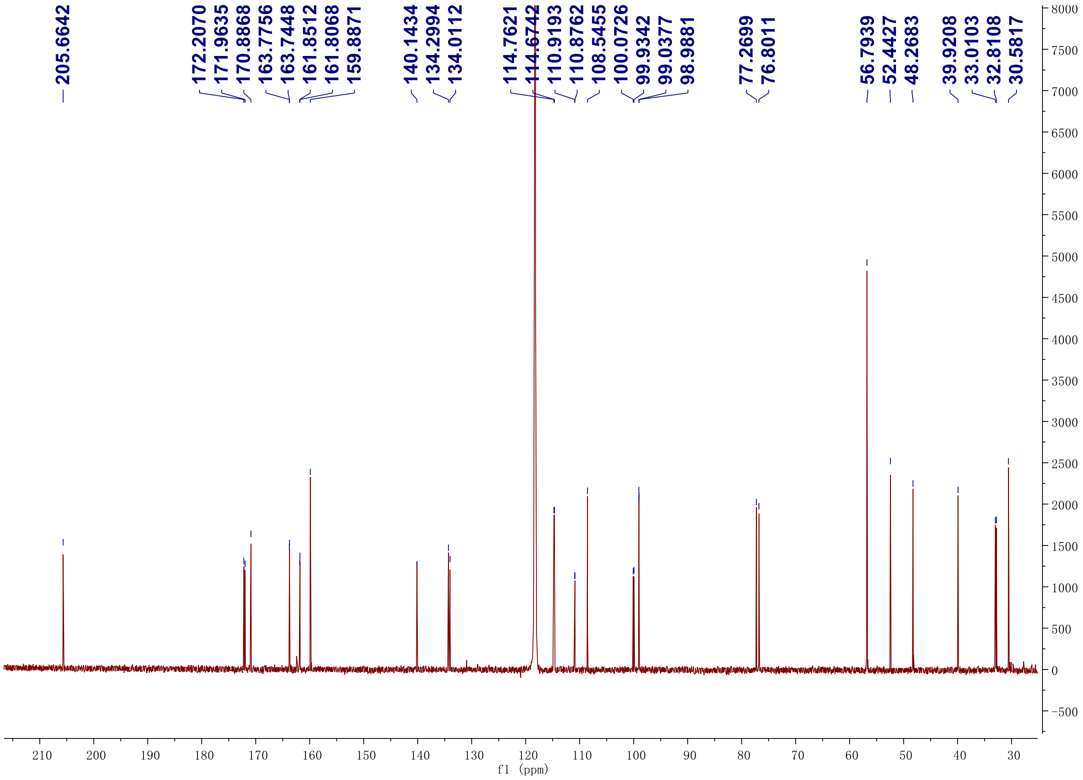

Figure S17. DEPT-135 and DEPT-90 ^{13}C NMR spectrum (125 MHz) of 2 in acetonitrile-d_3

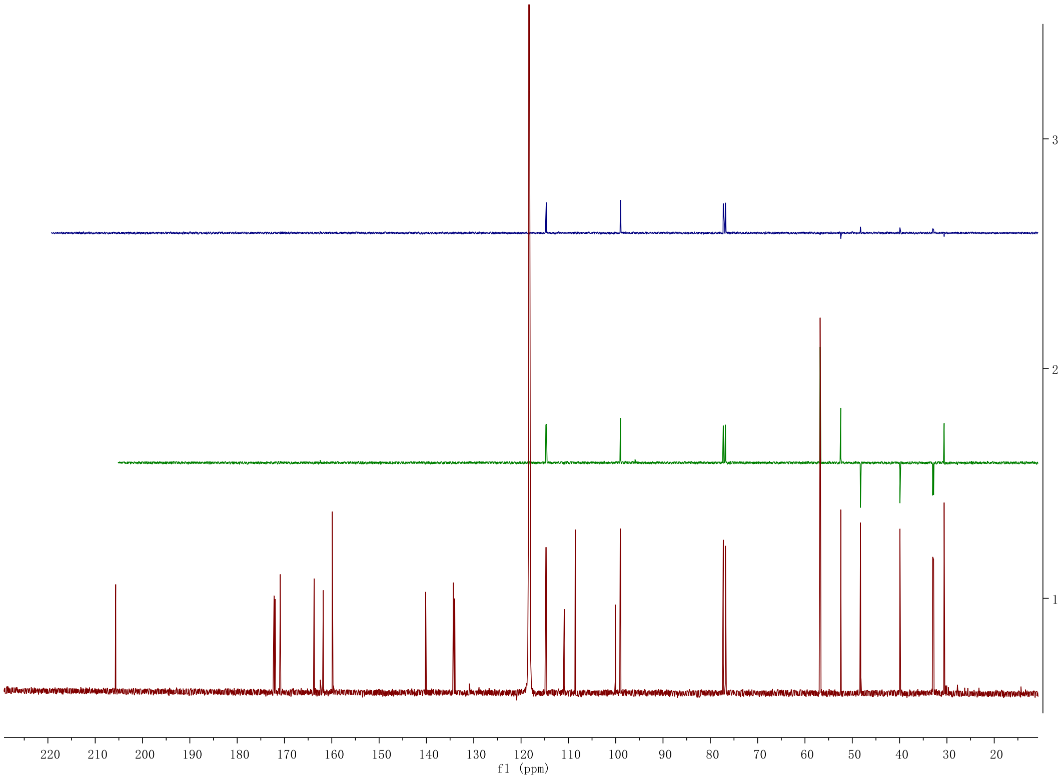

Figure S18. ^{1}H-^{1}H gCOSY NMR spectrum (500 MHz) of 2 in acetonitrile-d_3

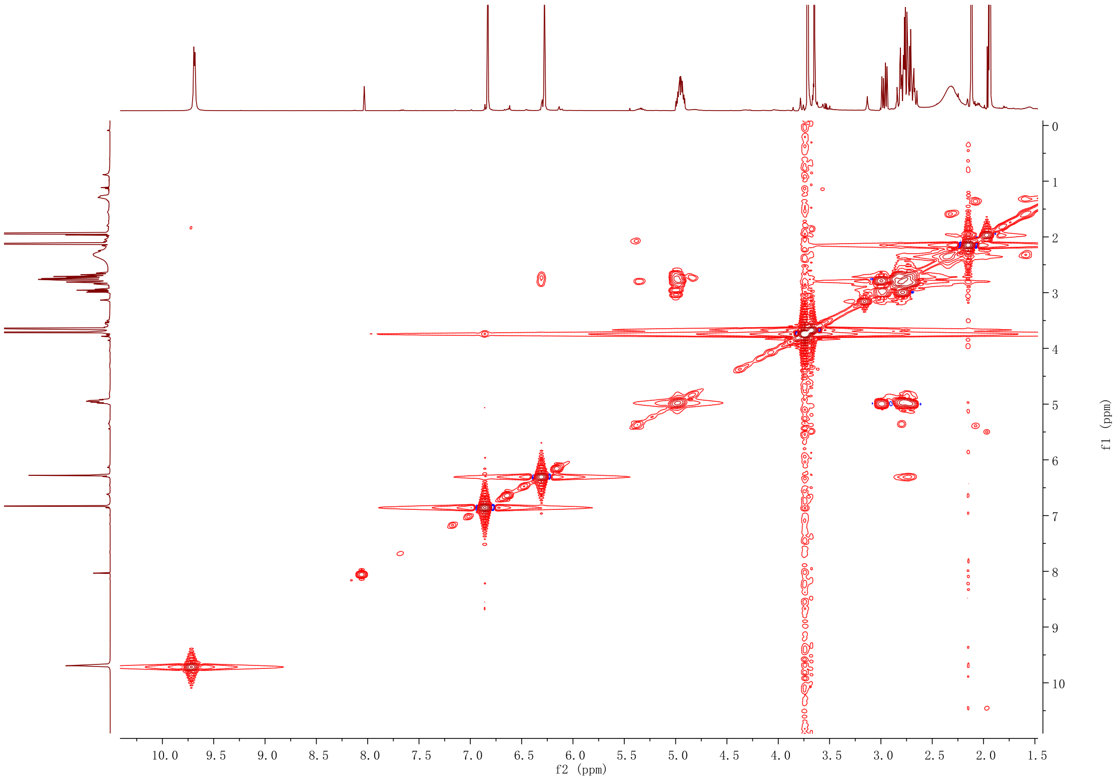

Figure S19. HSQC NMR spectrum (500 MHz) of 2 in acetonitrile-d_3

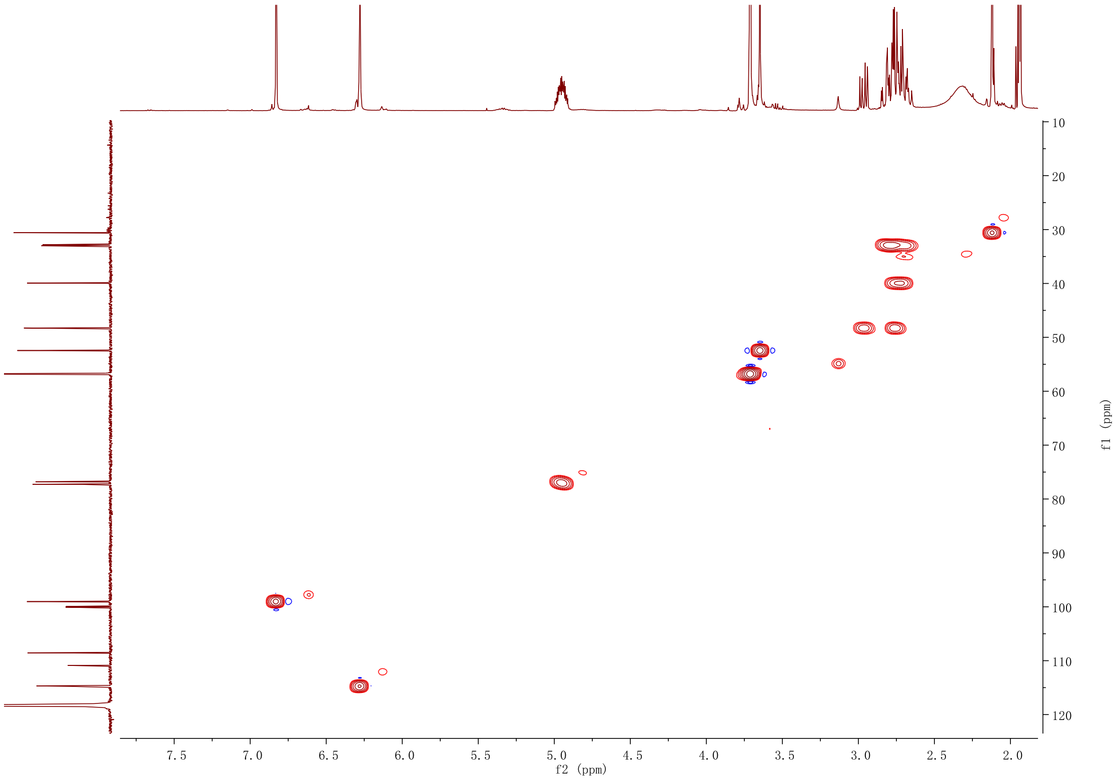

Figure S20. HMBC NMR spectrum (500 MHz) of 2 in acetonitrile-d_3

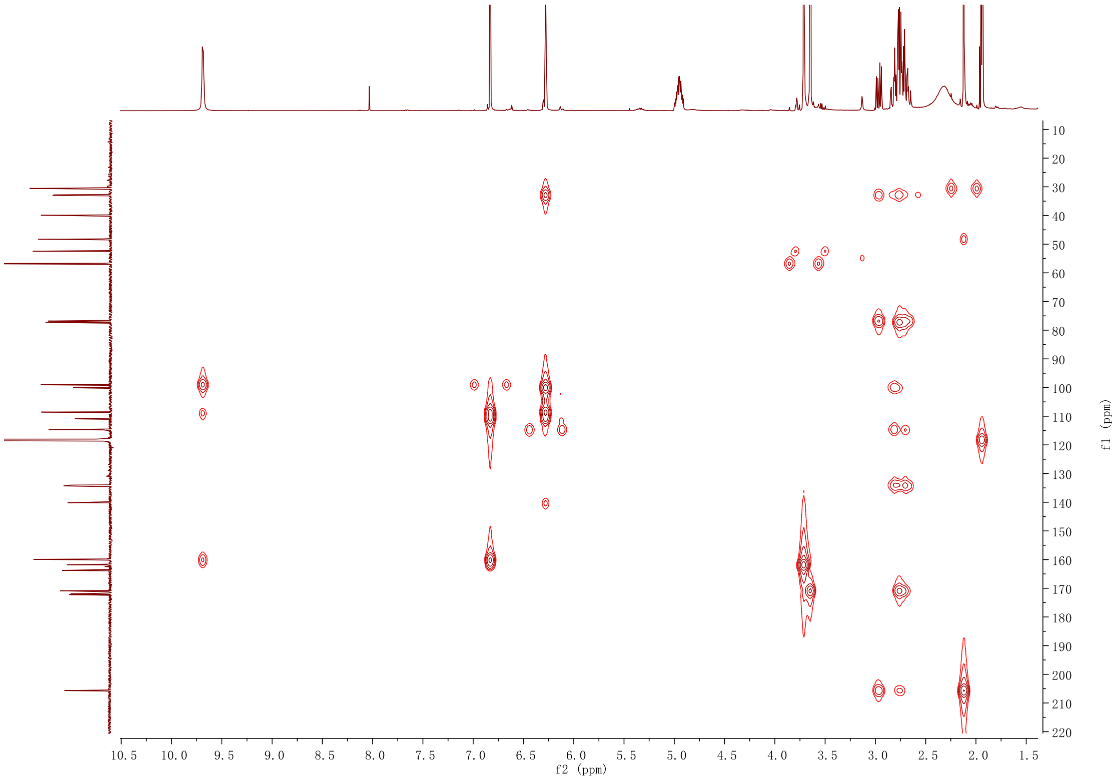

Figure S21. ^{1}H NMR spectrum (600 MHz) of 3 in acetonitrile-d_3

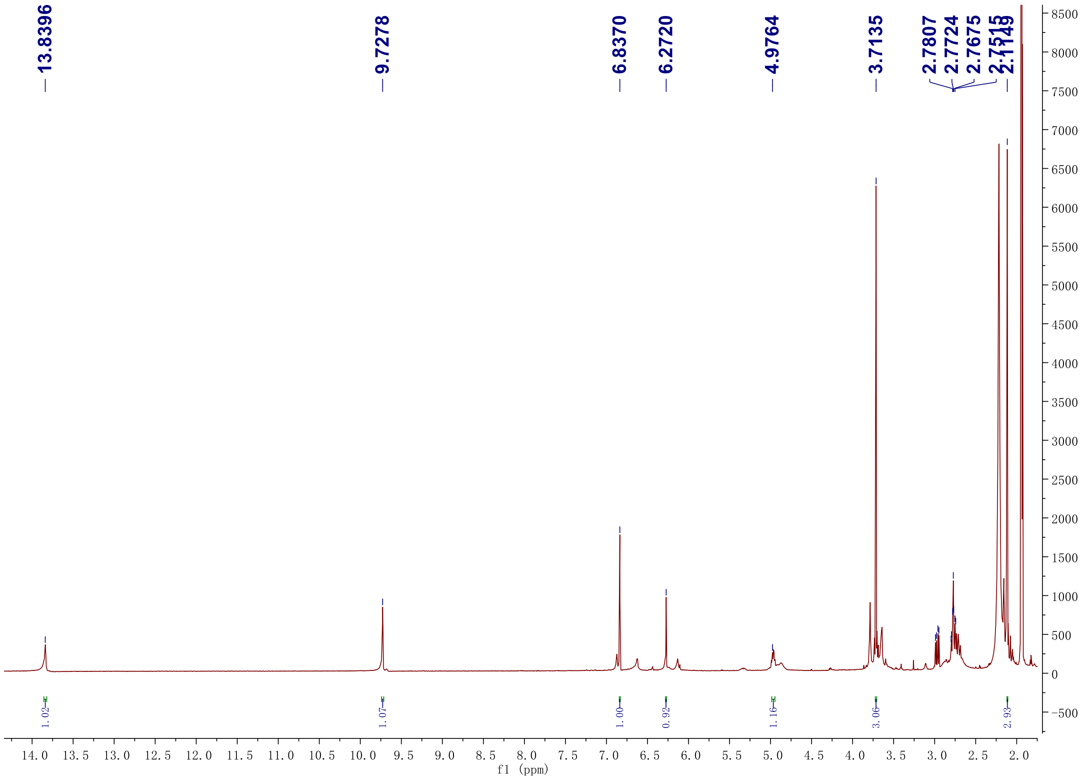

Figure S22. ^{13}C NMR spectrum (150 MHz) of 3 in acetonitrile-d_3

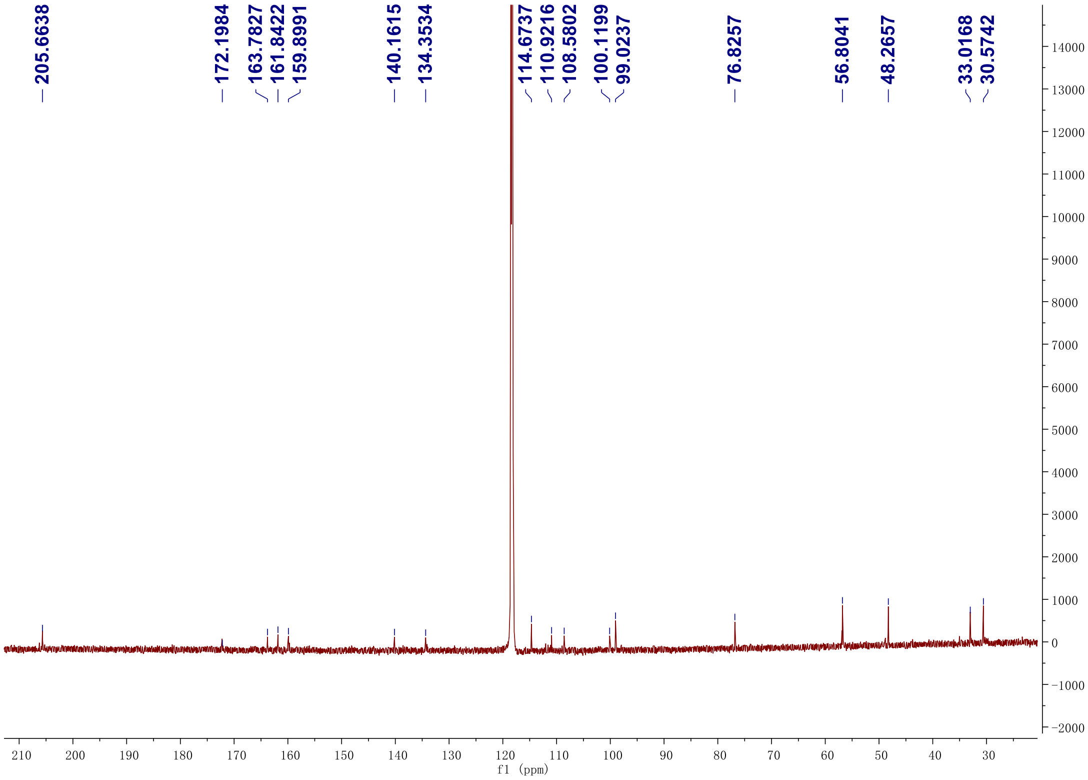

Figure S23. DEPT-135 ^{13}C NMR spectrum (150 MHz) of 3 in acetonitrile-d_3

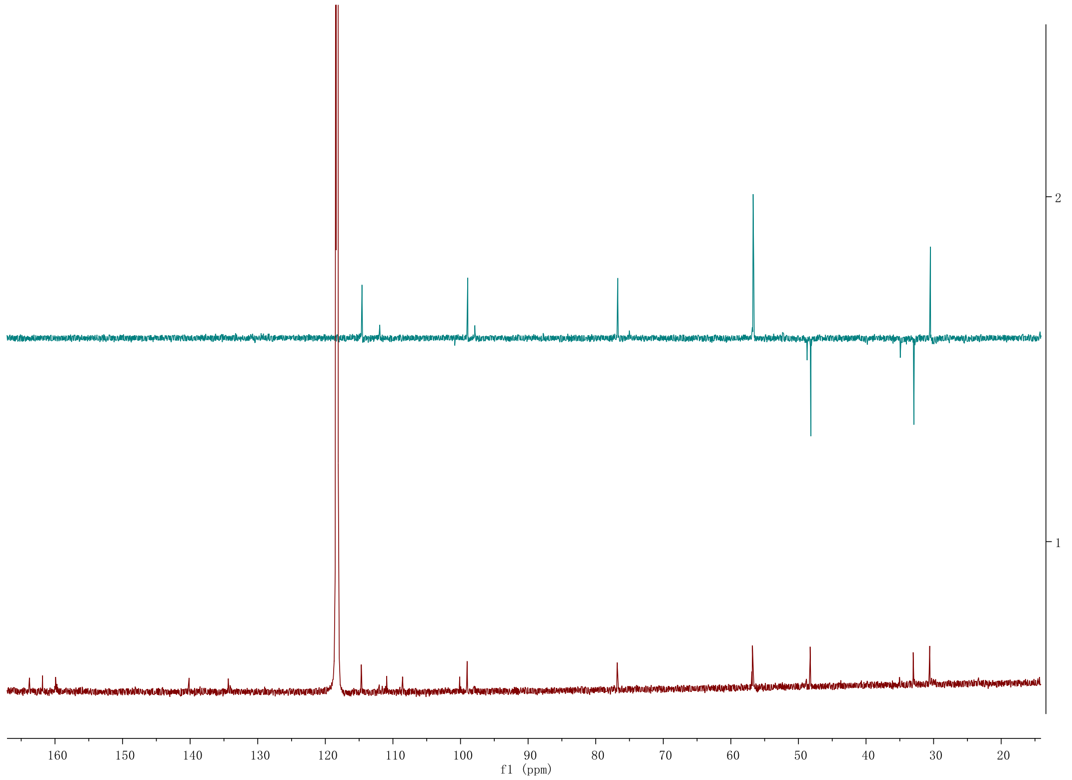

Figure S24. ^1H-^1H gCOSY NMR spectrum (600 MHz) of 3 in acetonitrile-*d_3*

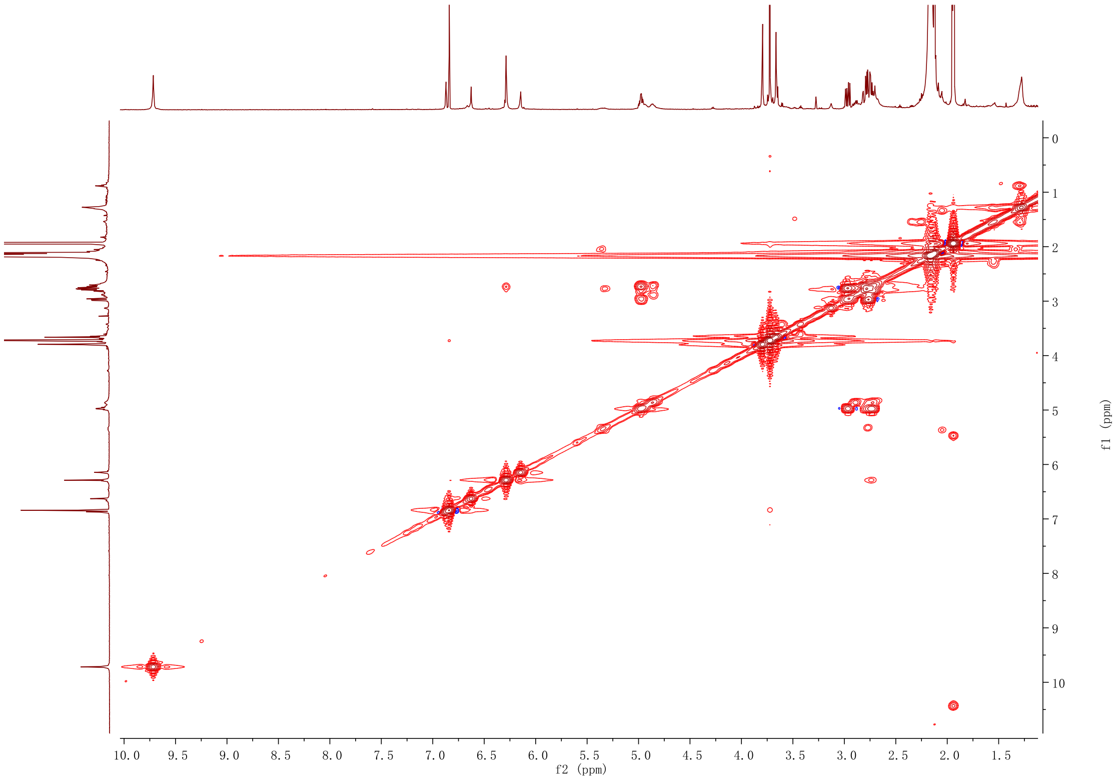

Figure S25. HSQC NMR spectrum (600 MHz) of 3 in acetonitrile-d_3

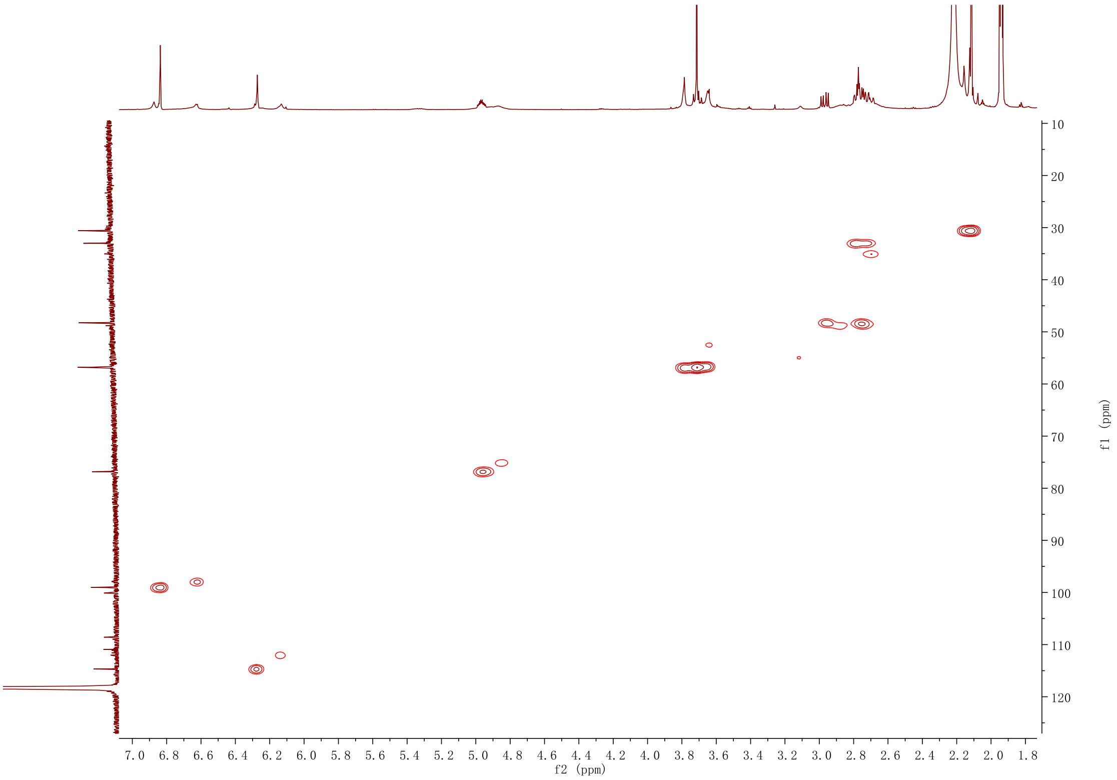

Figure S26. HMBC NMR spectrum (600 MHz) of 3 in acetonitrile-d_3

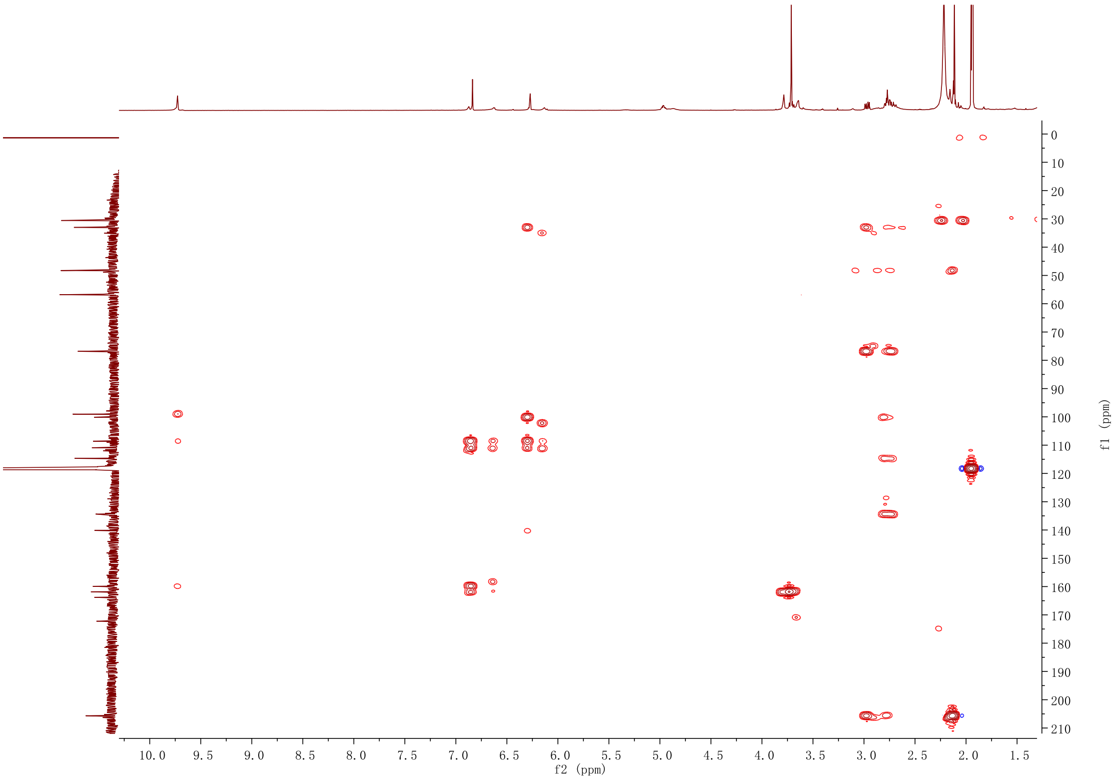

Figure S27. ^{1}H NMR spectrum (500 MHz) of 4 in acetonitrile-d_3

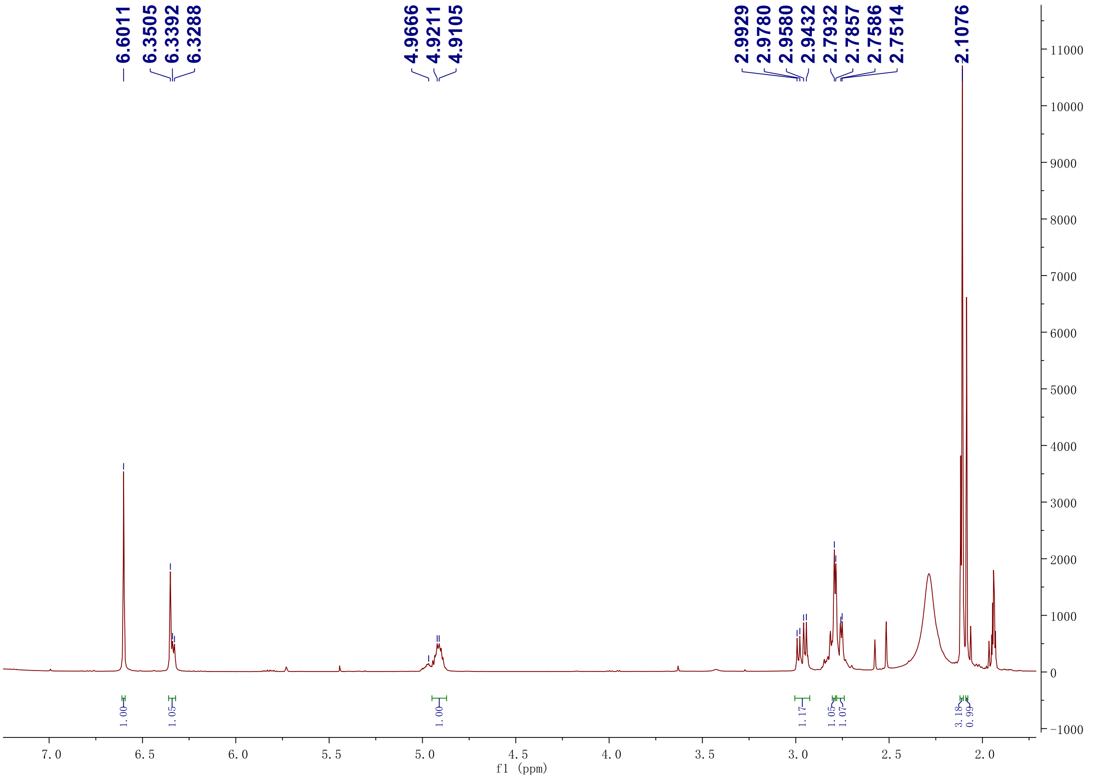

Figure S28. ^{13}C NMR spectrum (125 MHz) of 4 in acetonitrile-d_3

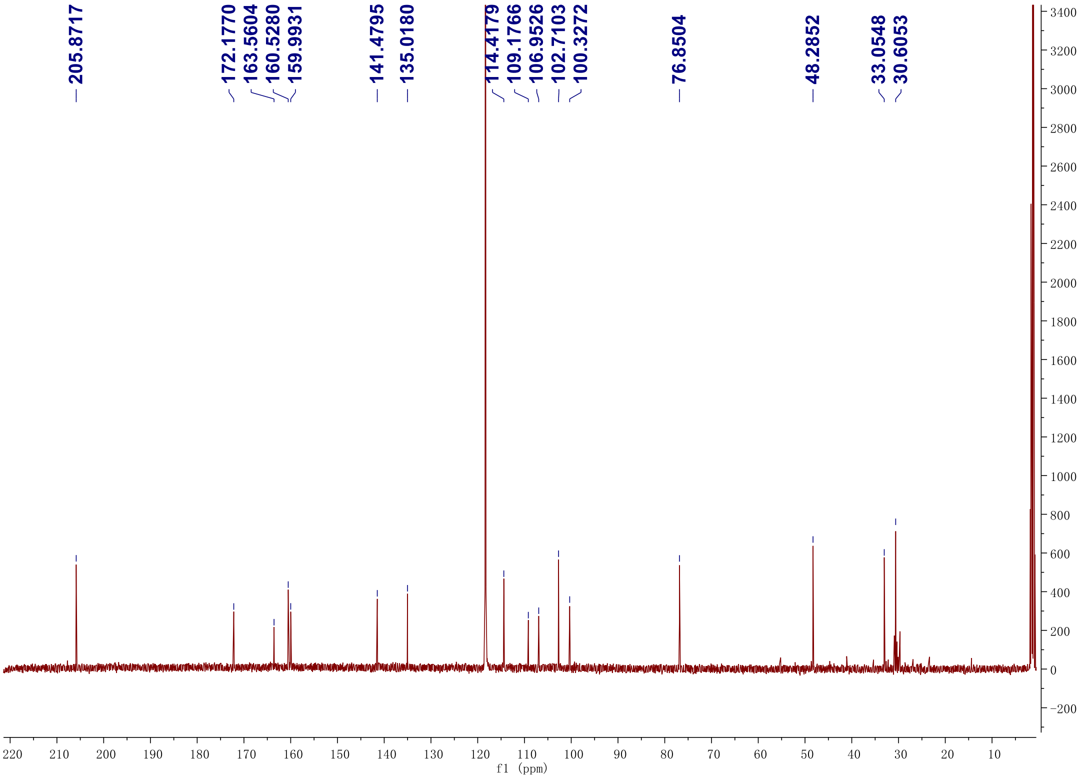

Figure S29. DEPT-135 ^{13}C NMR spectrum (125 MHz) of 4 in acetonitrile-d_3

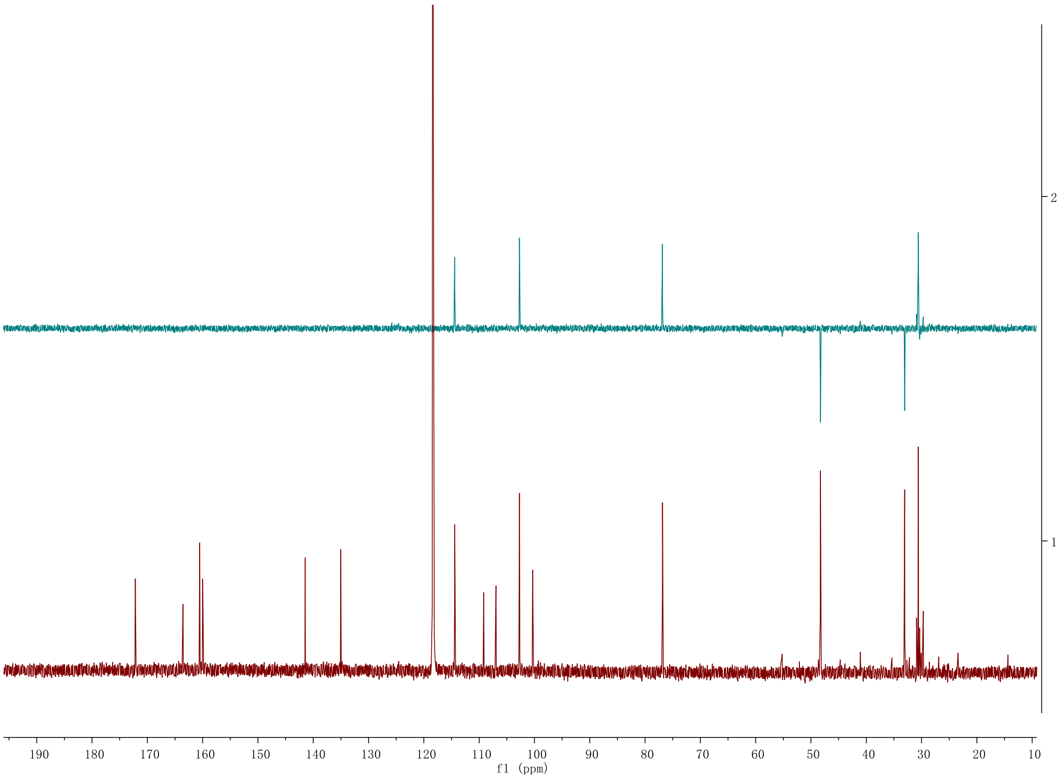

Figure S30. ^1H-^1H gCOSY NMR spectrum (500 MHz) of 4 in acetonitrile-d_3

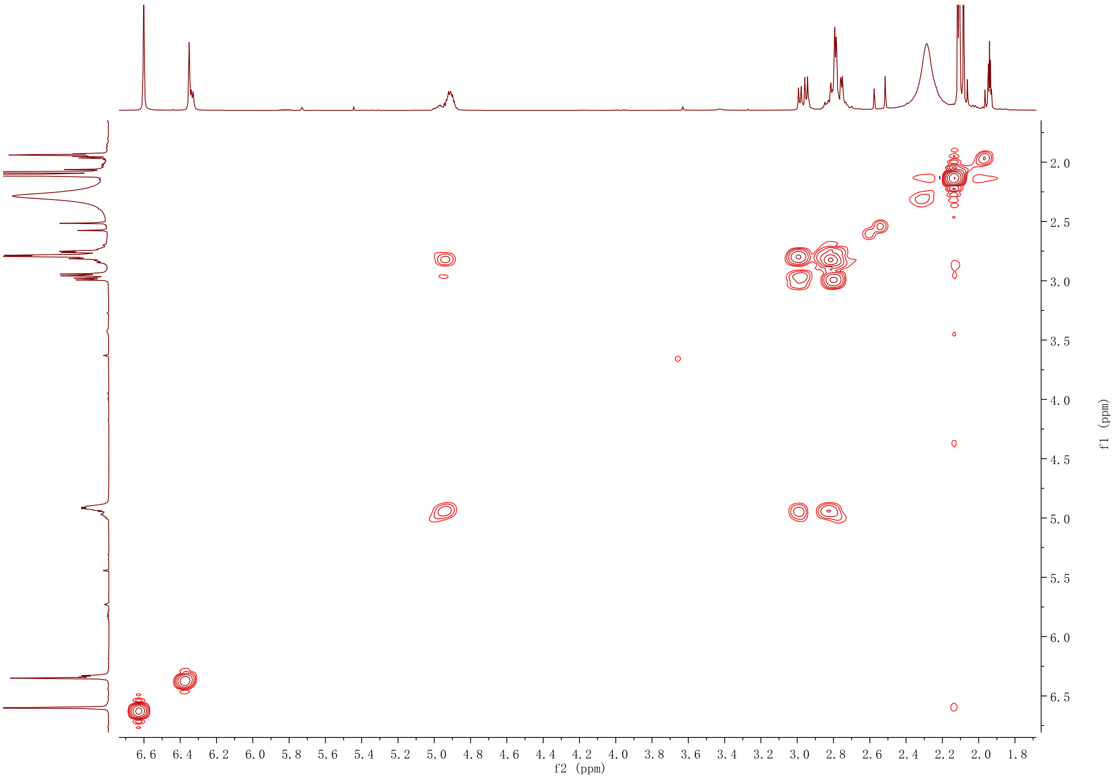

Figure S31. HSQC NMR spectrum (500 MHz) of 4 in acetonitrile-d_3

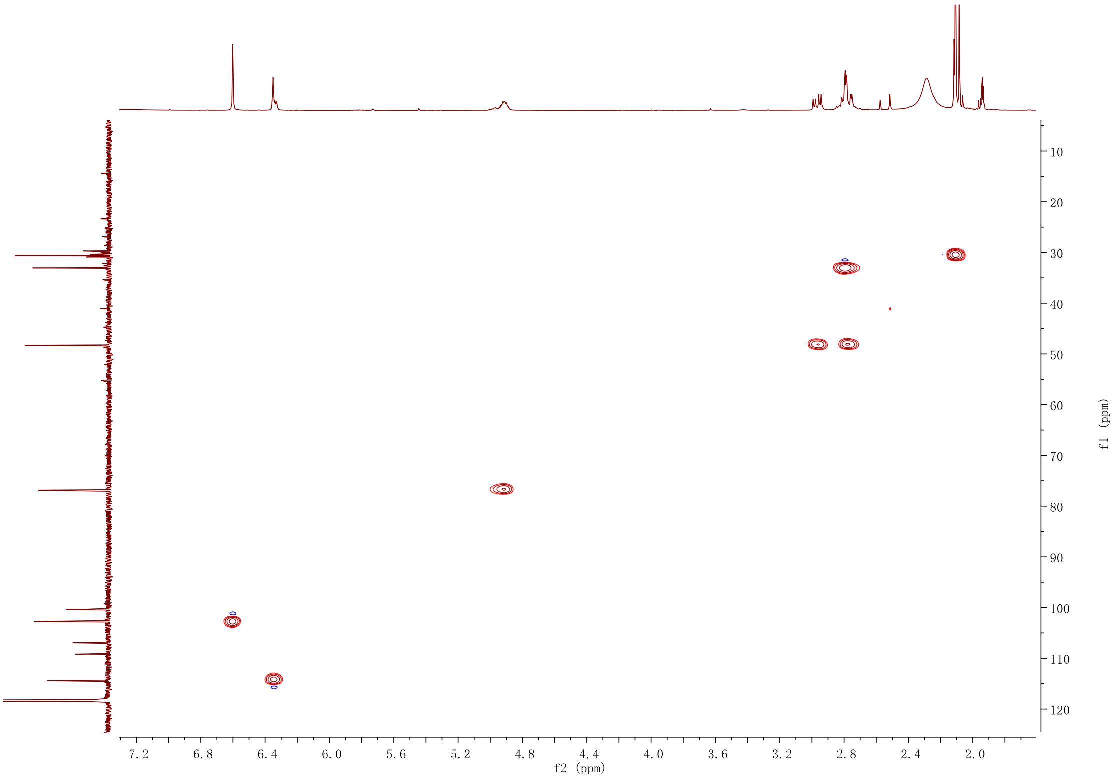

Figure S32. HMBC NMR spectrum (500 MHz) of 4 in acetonitrile-d_3

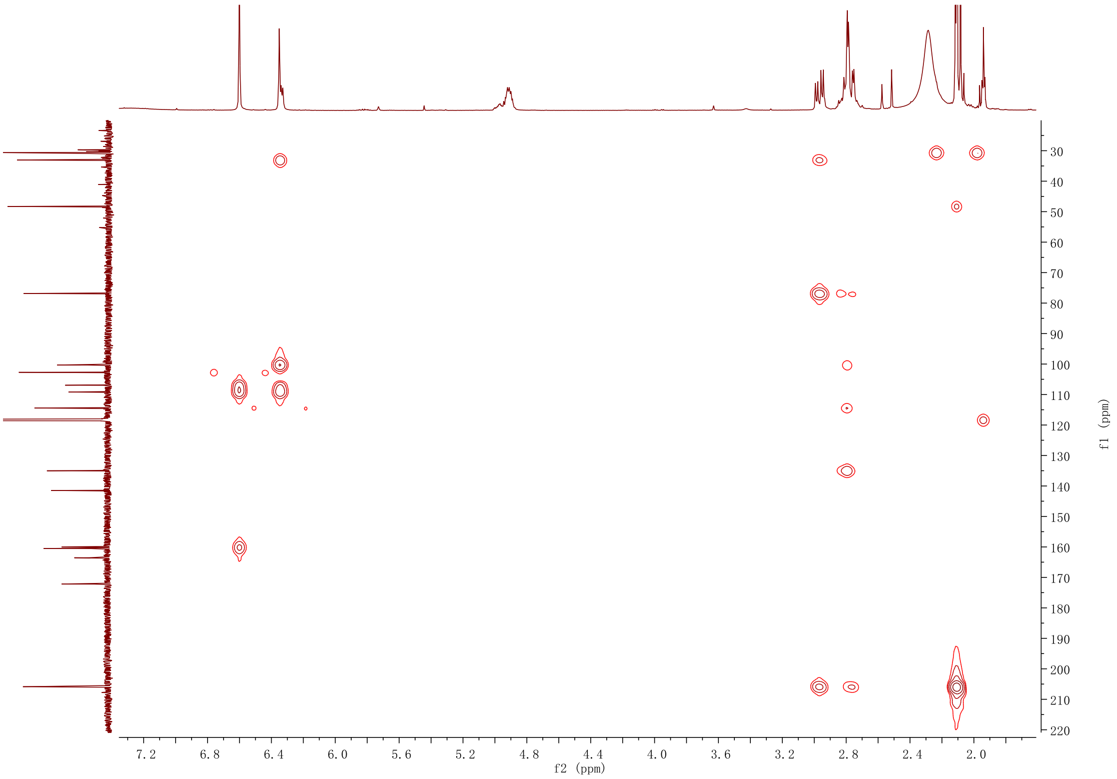

Figure S33. ^{1}H NMR spectrum (600 MHz) of 5 in acetonitrile-d_3

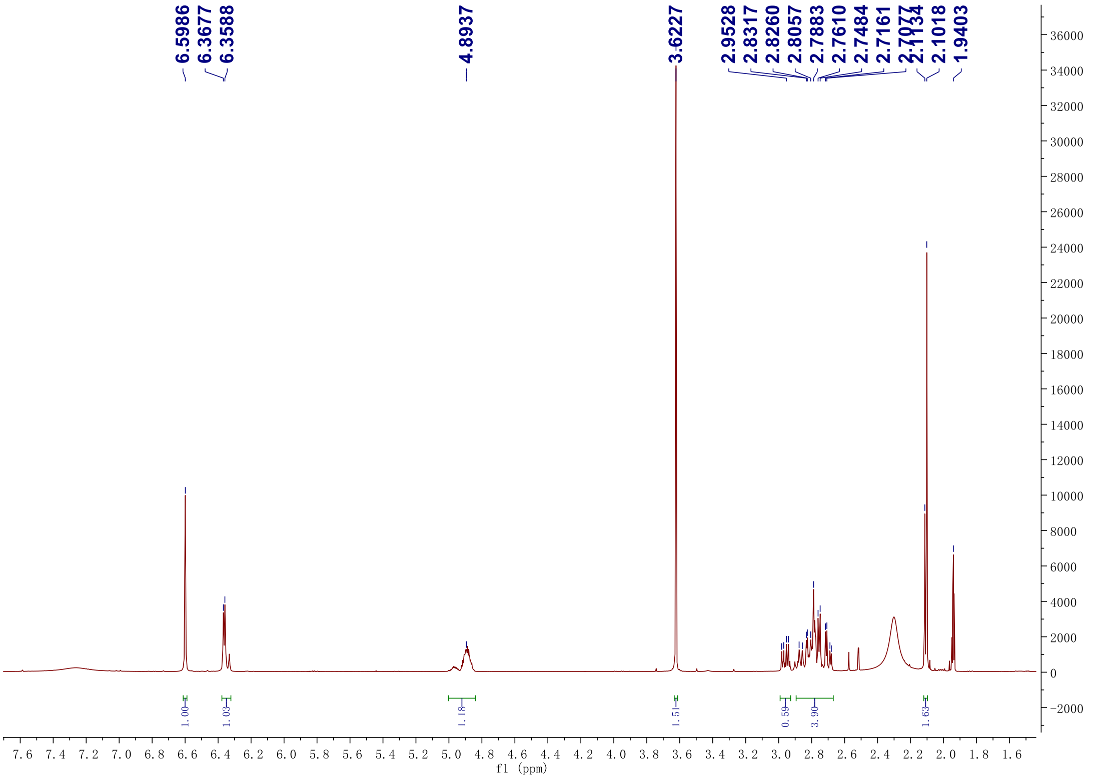

Figure S34. ^{13}C NMR spectrum (150 MHz) of 5 in acetonitrile-d_3

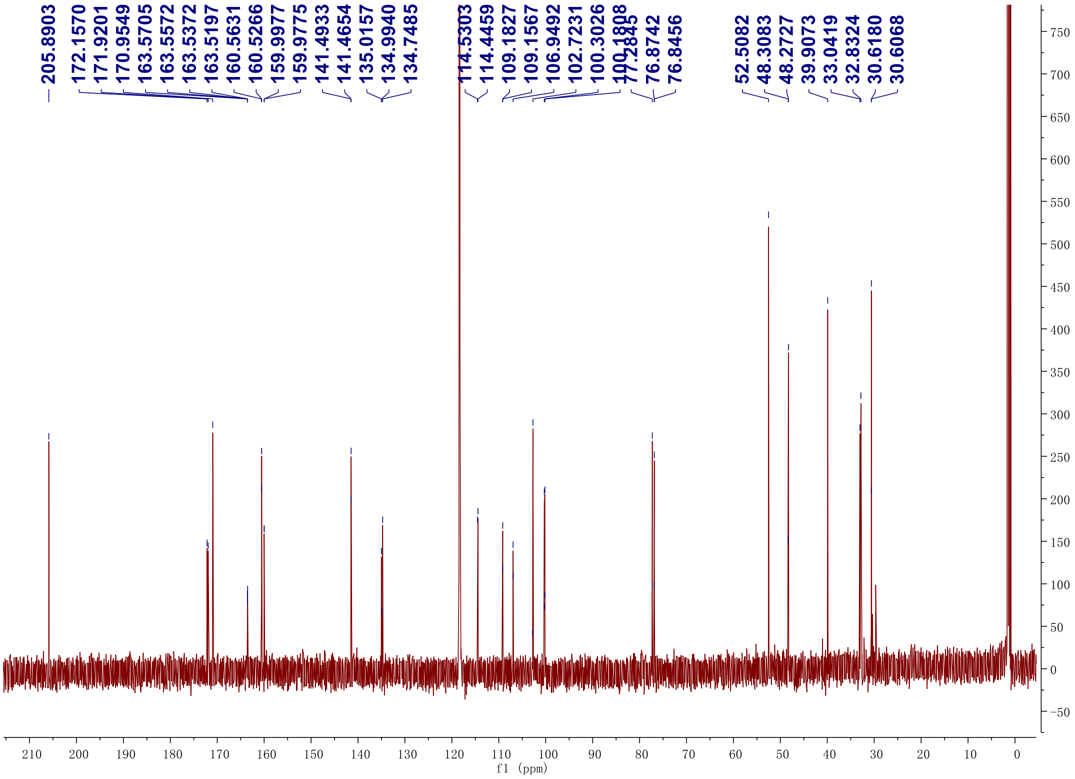

Figure S35. DEPT-135 ^{13}C NMR spectrum (150 MHz) of 5 in acetonitrile-d_3

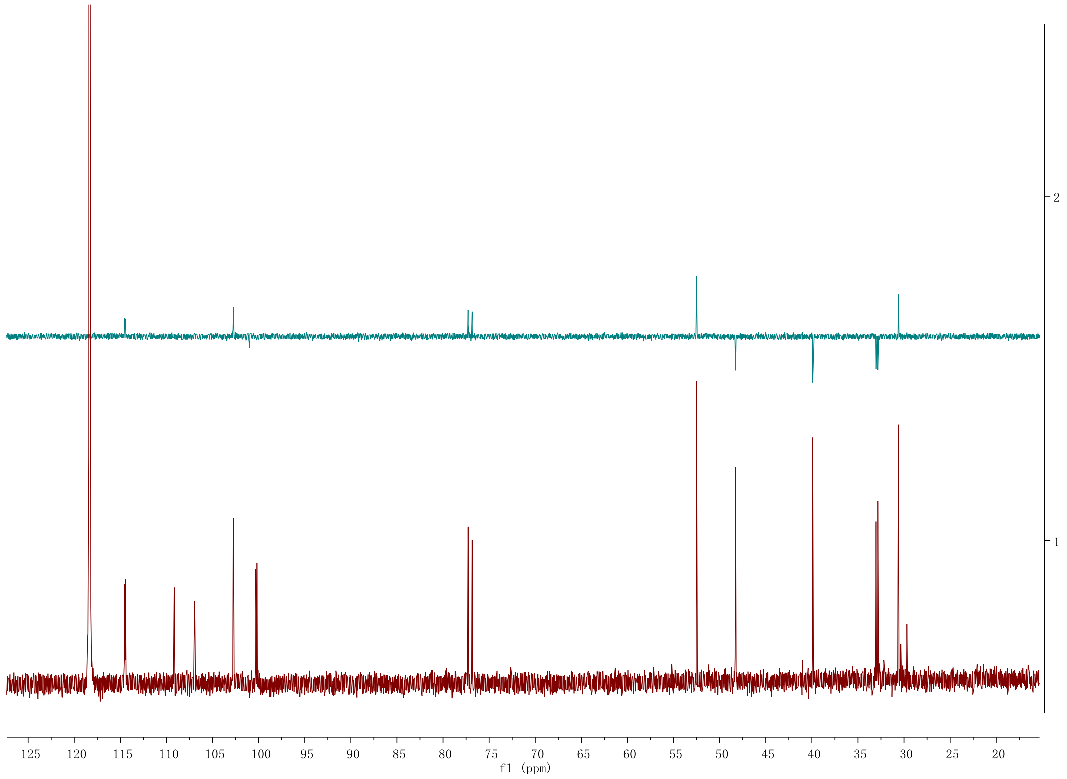

Figure S36. ^1H-^1H gCOSY NMR spectrum (500 MHz) of 5 in acetonitrile-d_3

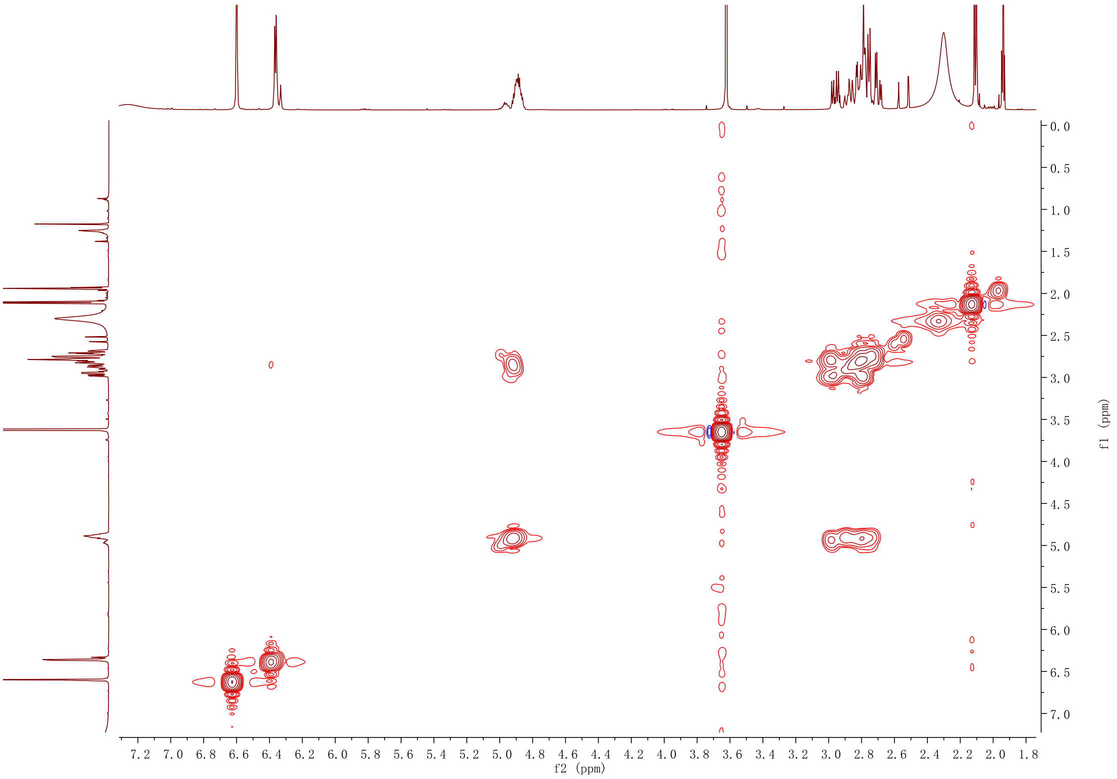

Figure S37. HSQC NMR spectrum (600 MHz) of 5 in acetonitrile-d_3

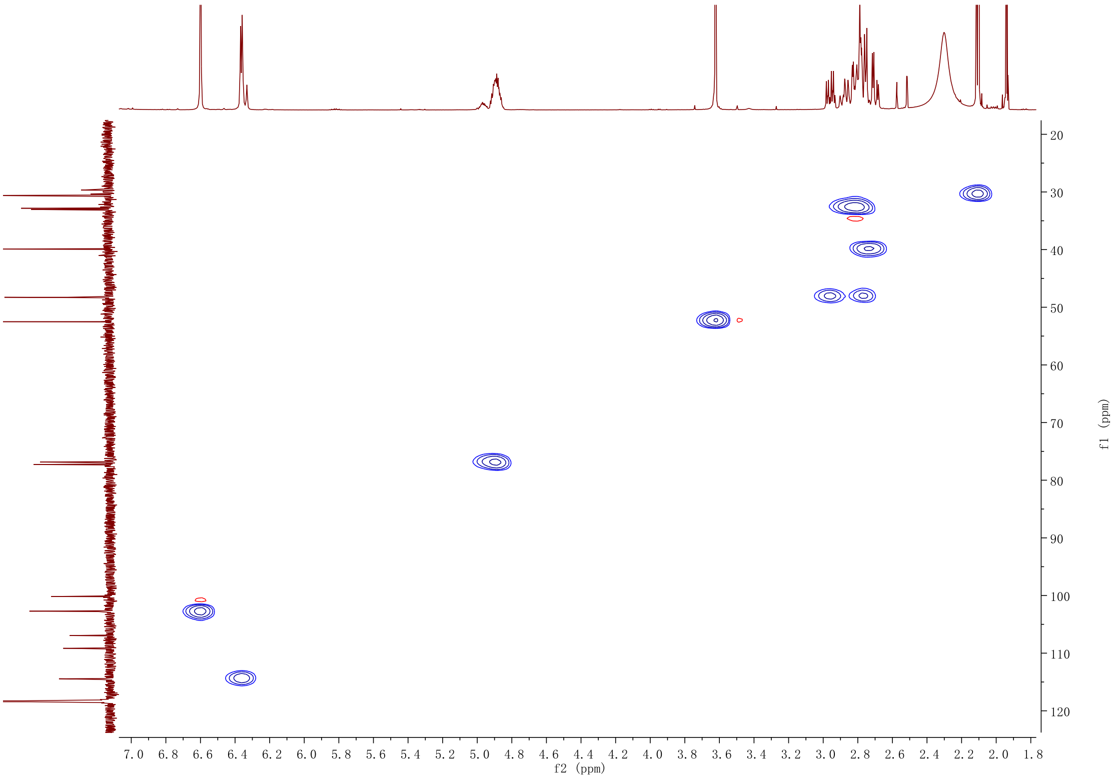

Figure S38. HMBC NMR spectrum (600 MHz) of 5 in acetonitrile-d_3

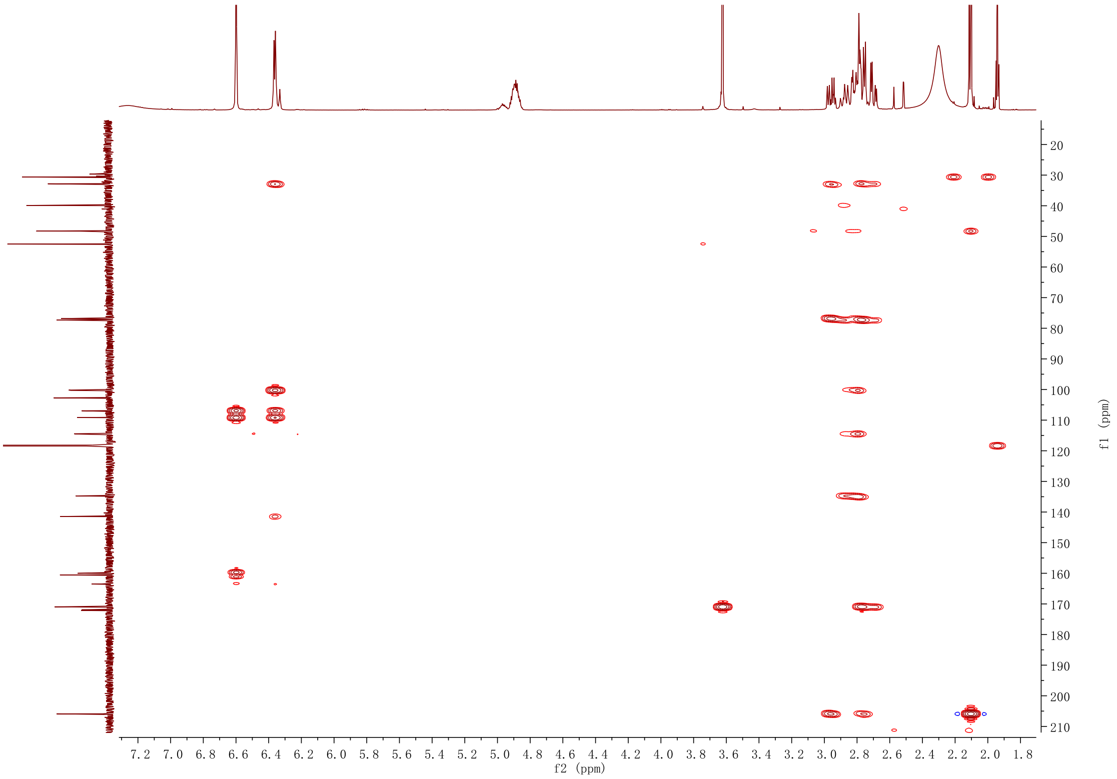

Figure S39. ^{1}H NMR spectrum (500 MHz) of 7 in chloroform-*d*

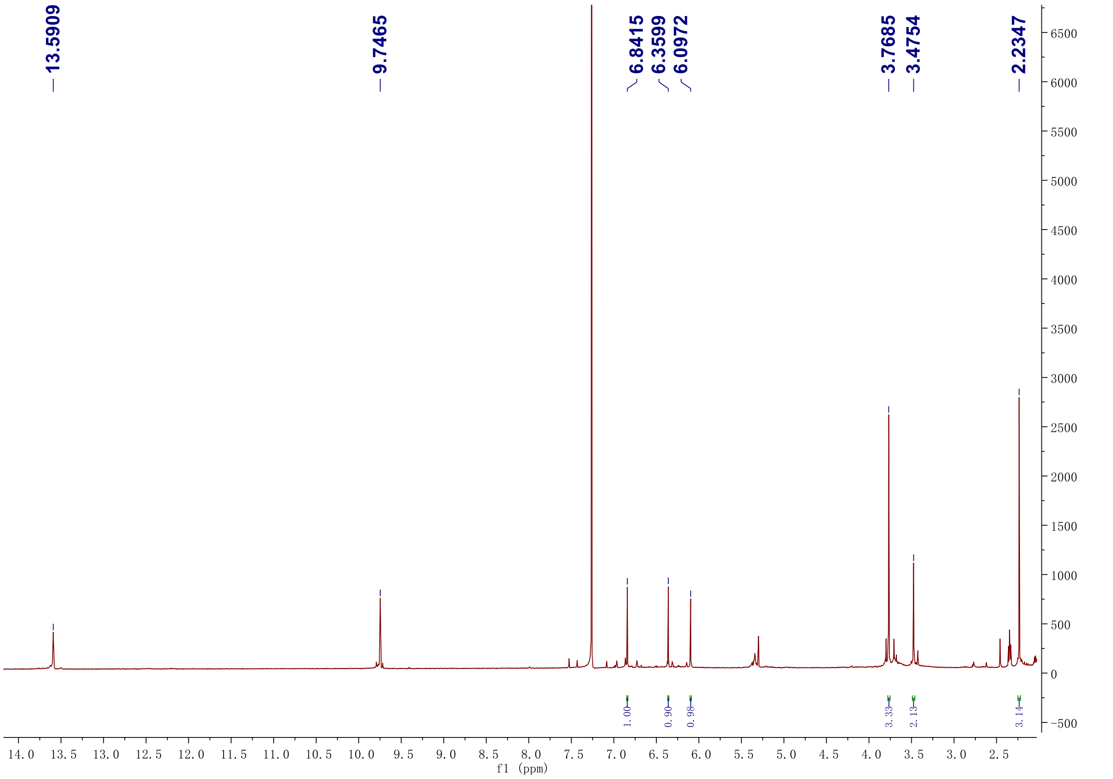

Figure S40. ^{13}C NMR spectrum (125 MHz) of 7 in chloroform-*d*

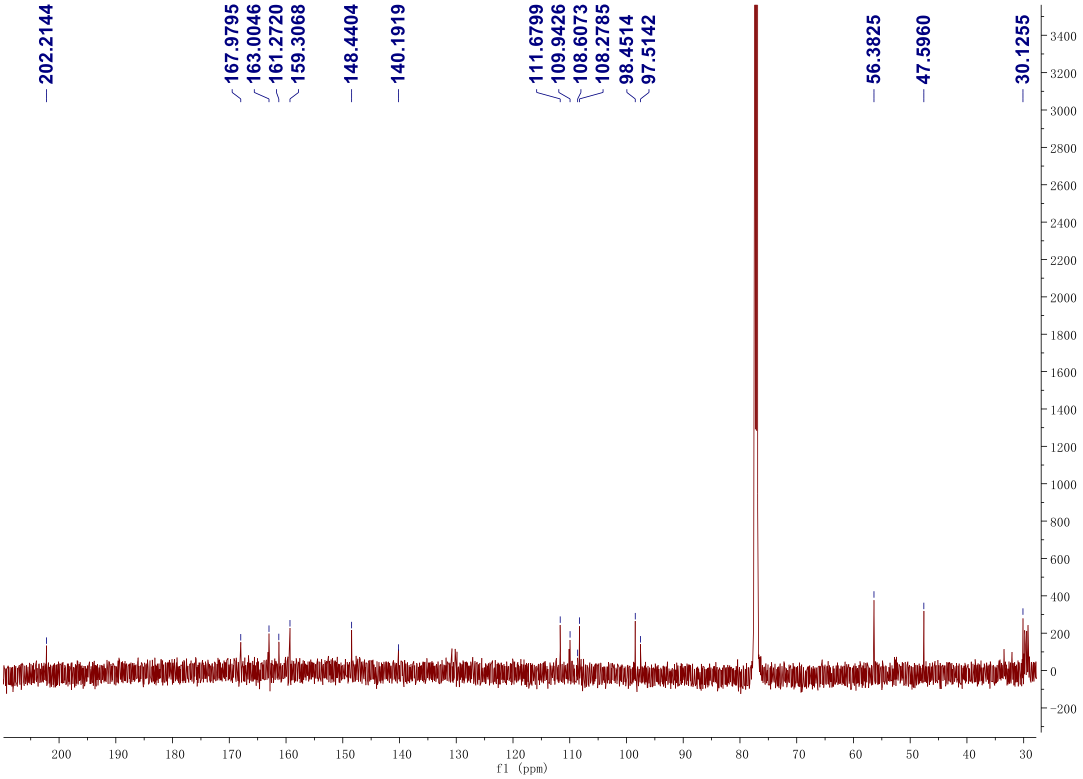

Figure S41. DEPT-135 ^{13}C NMR spectrum (125 MHz) of 7 in chloroform-*d*

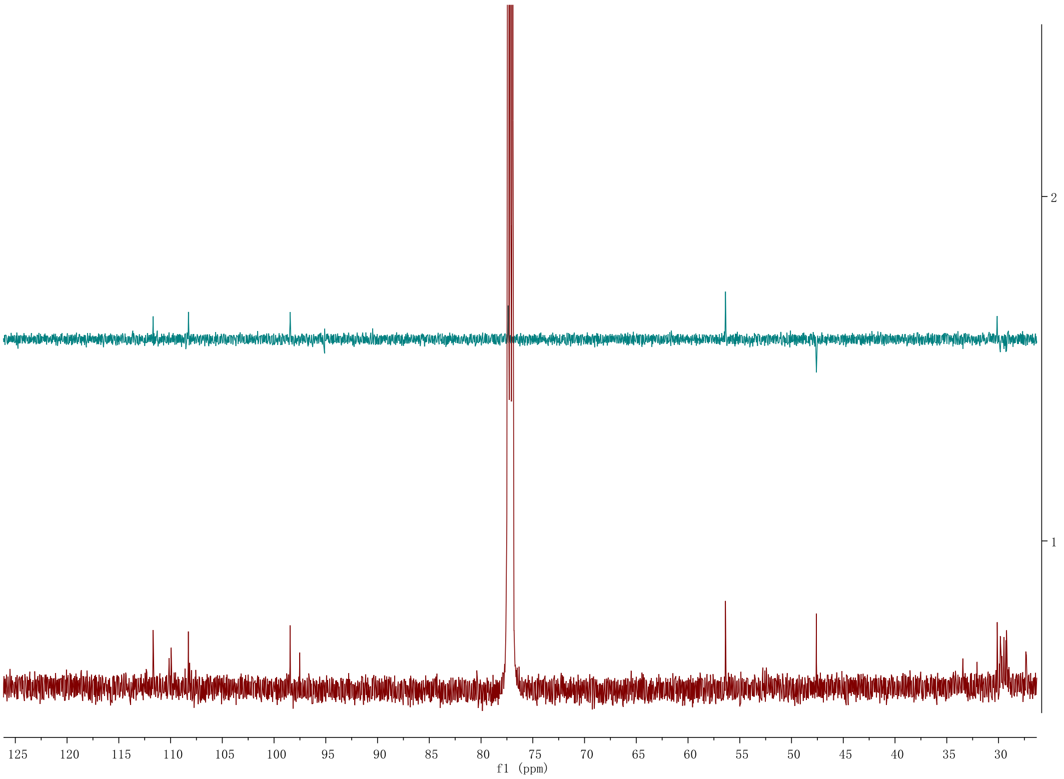

Figure S42. ^1H-^1H gCOSY NMR spectrum (500 MHz) of 7 in chloroform-*d*

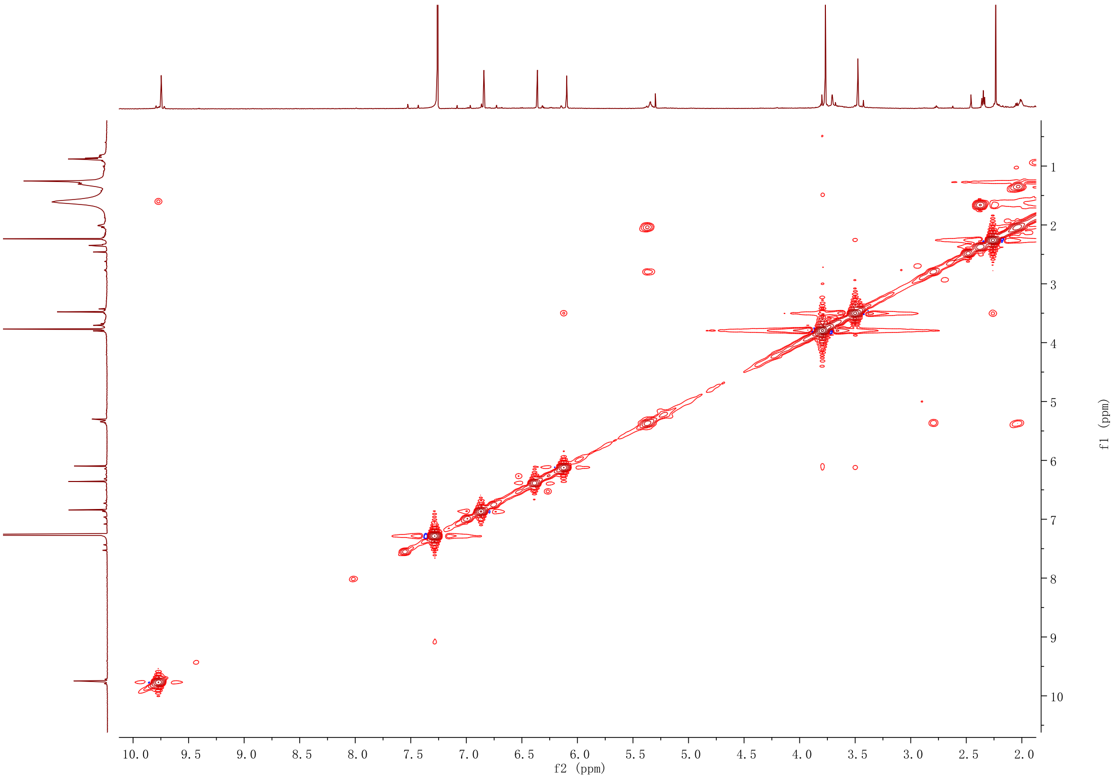

Figure S43. HSQC NMR spectrum (500 MHz) of 7 in chloroform-*d*

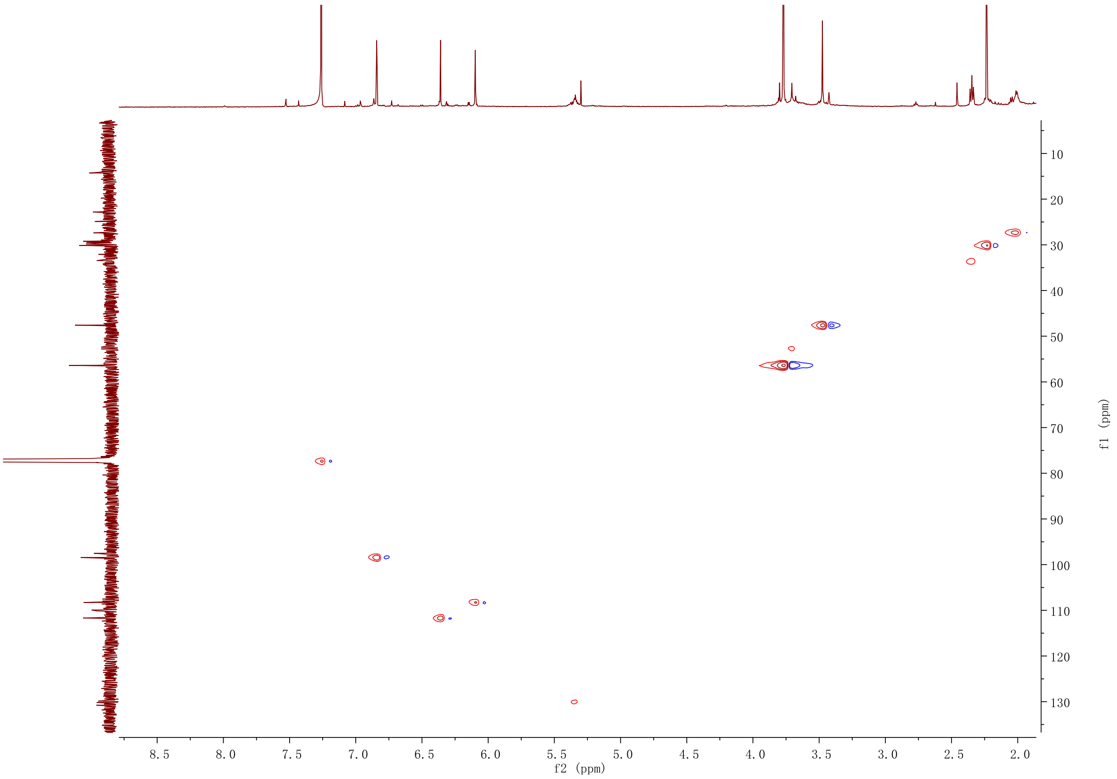

Figure S44. HMBC NMR spectrum (500 MHz) of 7 in chloroform-*d*

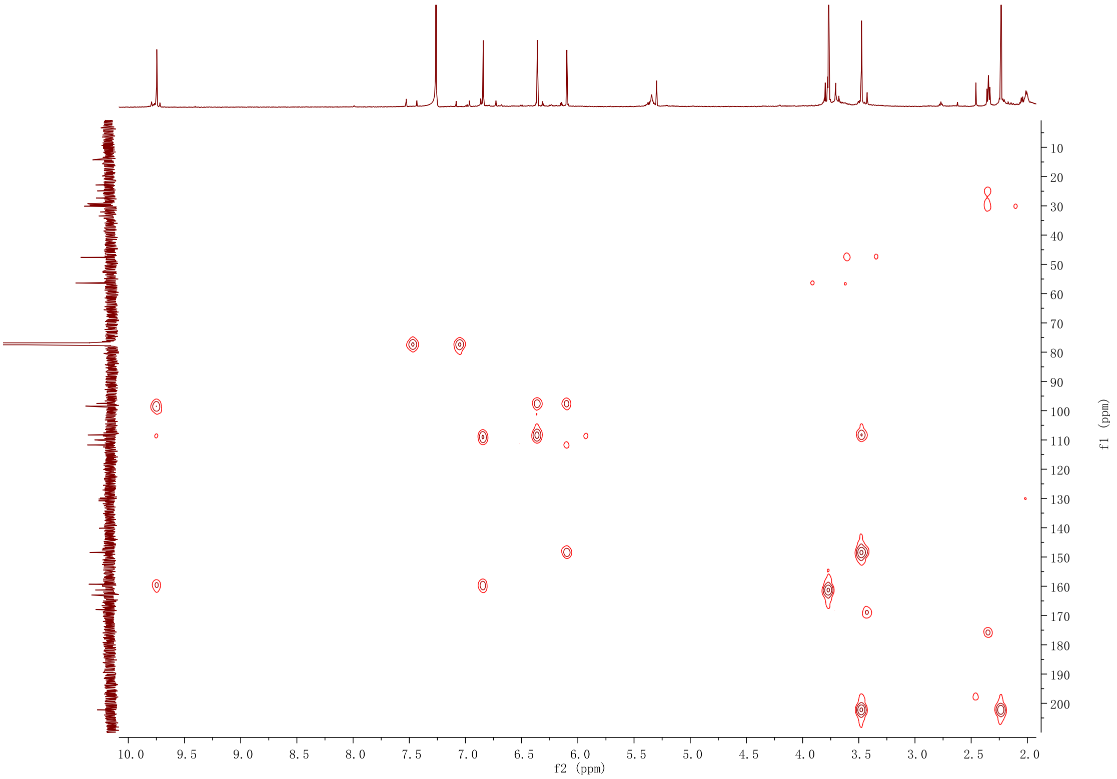

Figure S45. ¹H NMR spectrum (600 MHz) of 8 in chloroform-*d*

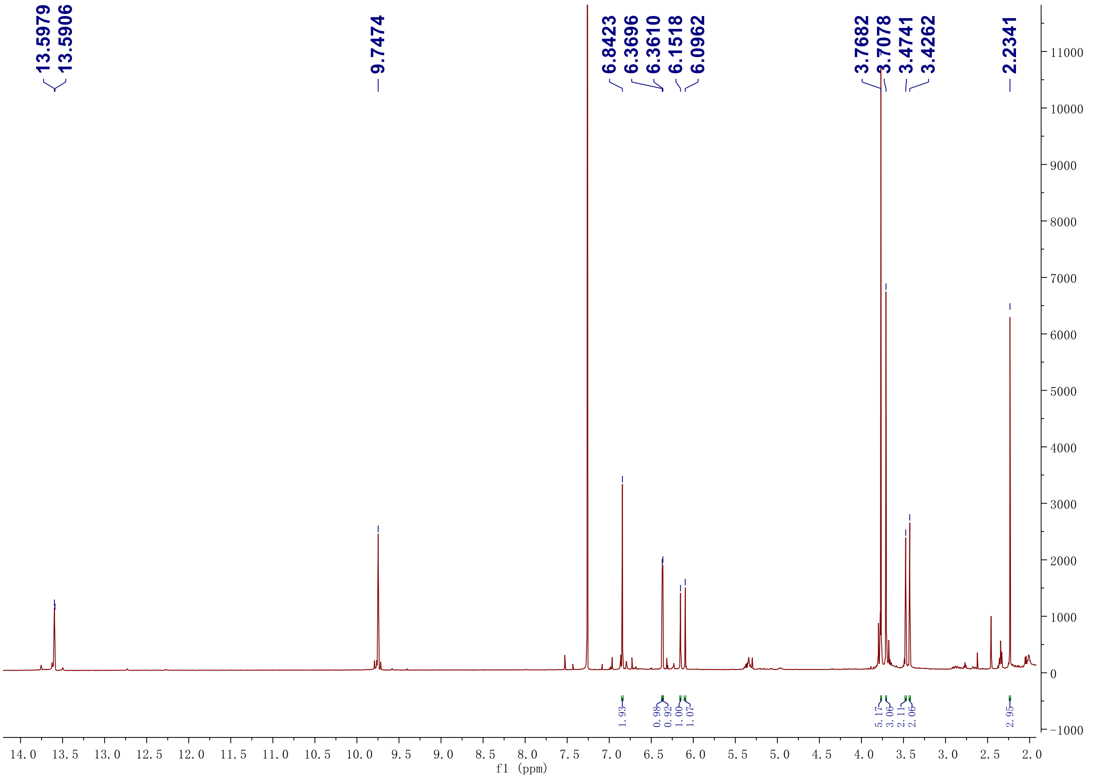

Figure S46. ^{13}C NMR spectrum (150 MHz) of 8 in chloroform-*d*

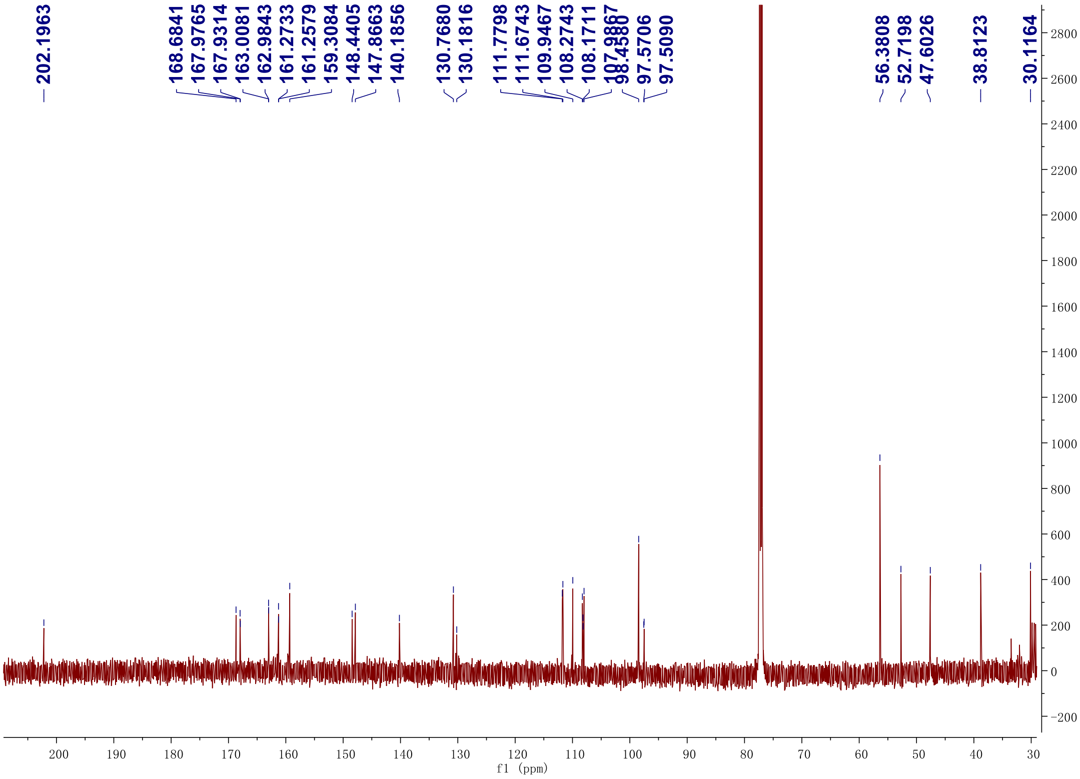

Figure S47. DEPT-135 ^{13}C NMR spectrum (150 MHz) of 8 in chloroform-*d*

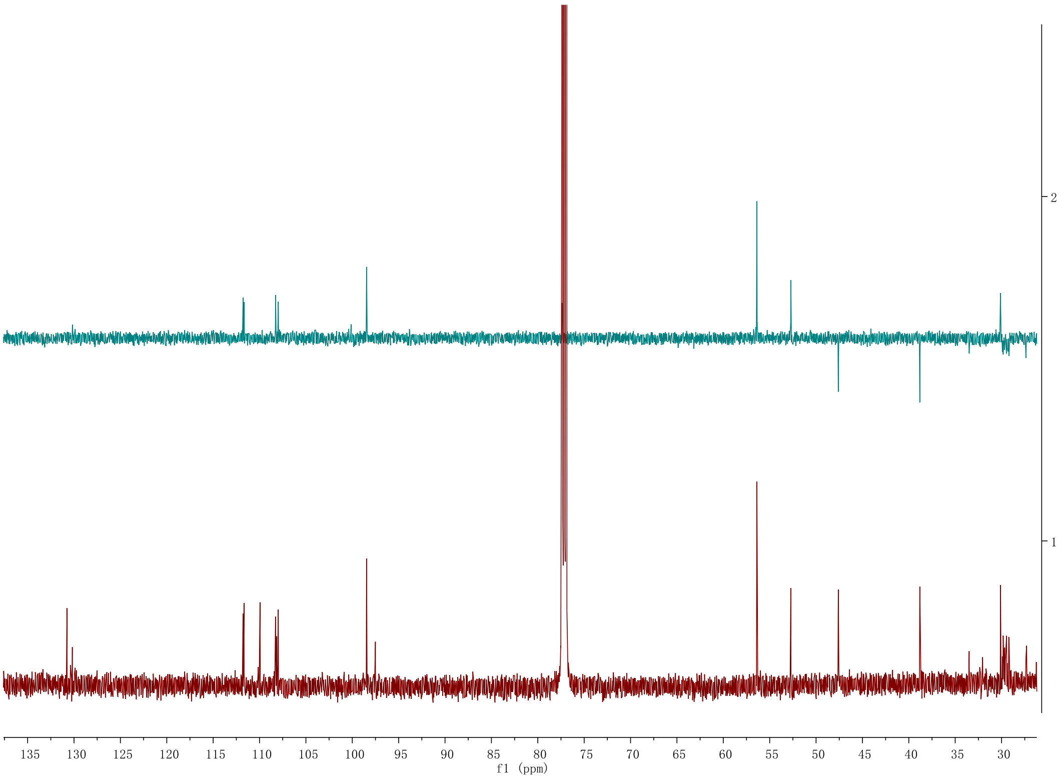

Figure S48. ^1H-^1H gCOSY NMR spectrum (500 MHz) of 8 in chloroform-*d*

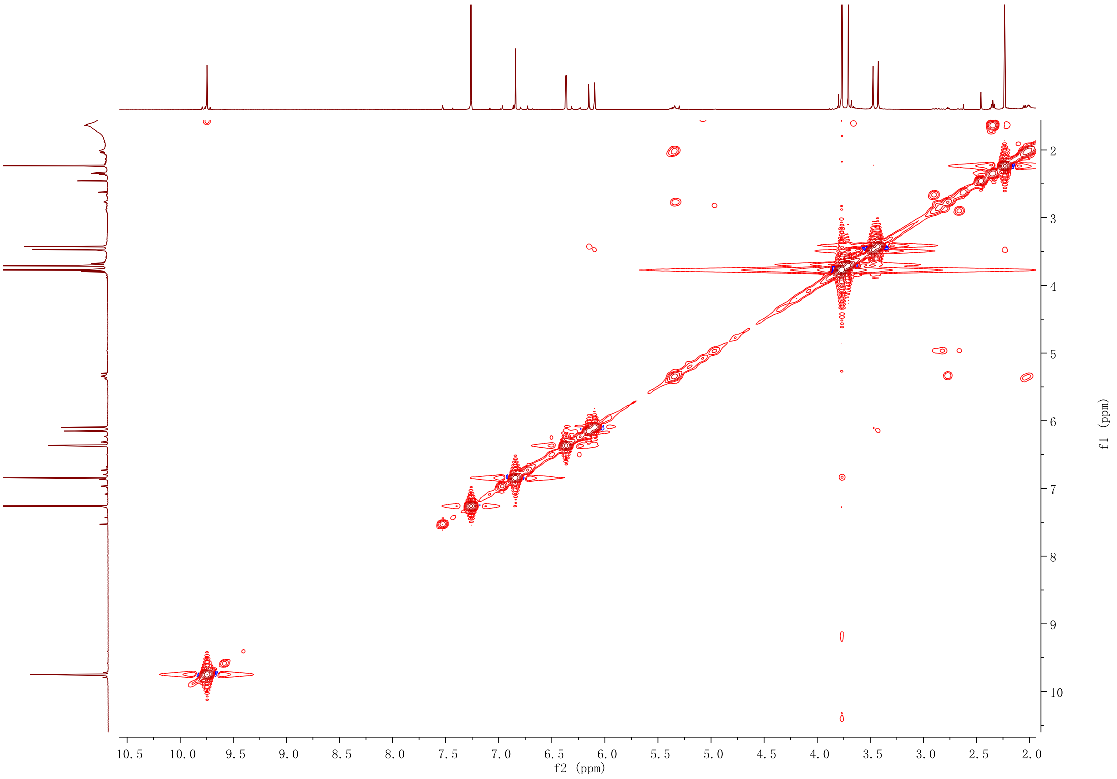

Figure S49. HSQC NMR spectrum (600 MHz) of 8 in chloroform-*d*

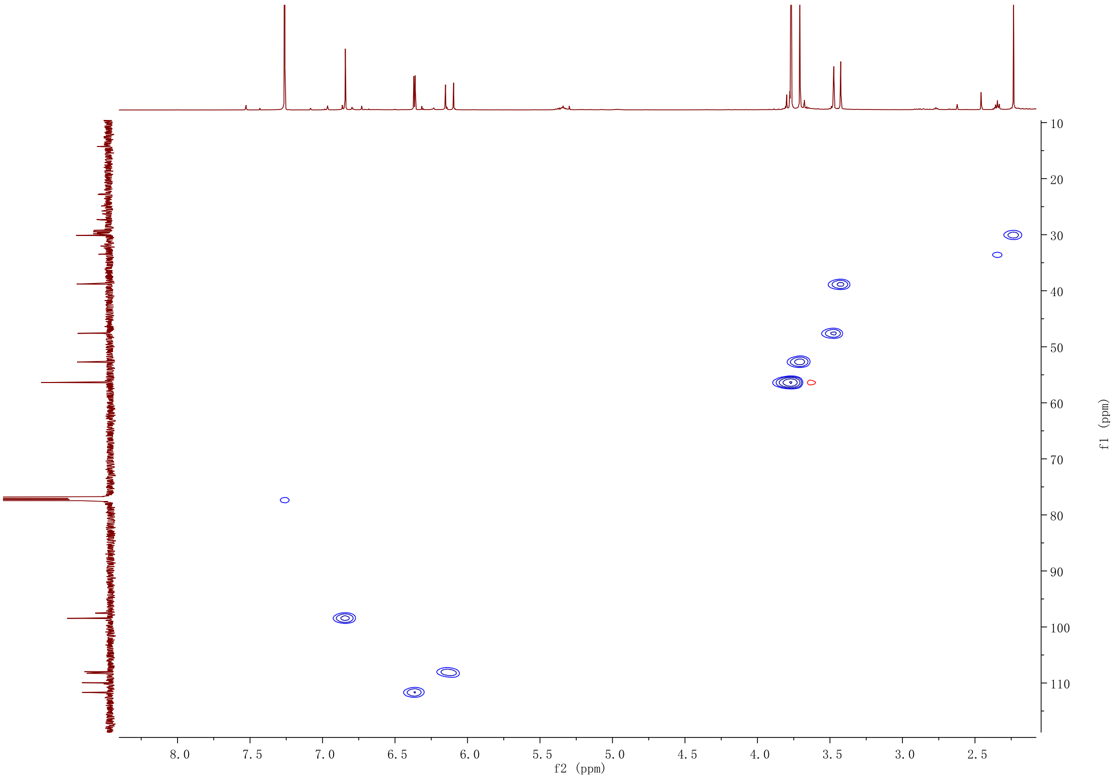

Figure S50 HMBC NMR spectrum (600 MHz) of 8 in chloroform-*d*

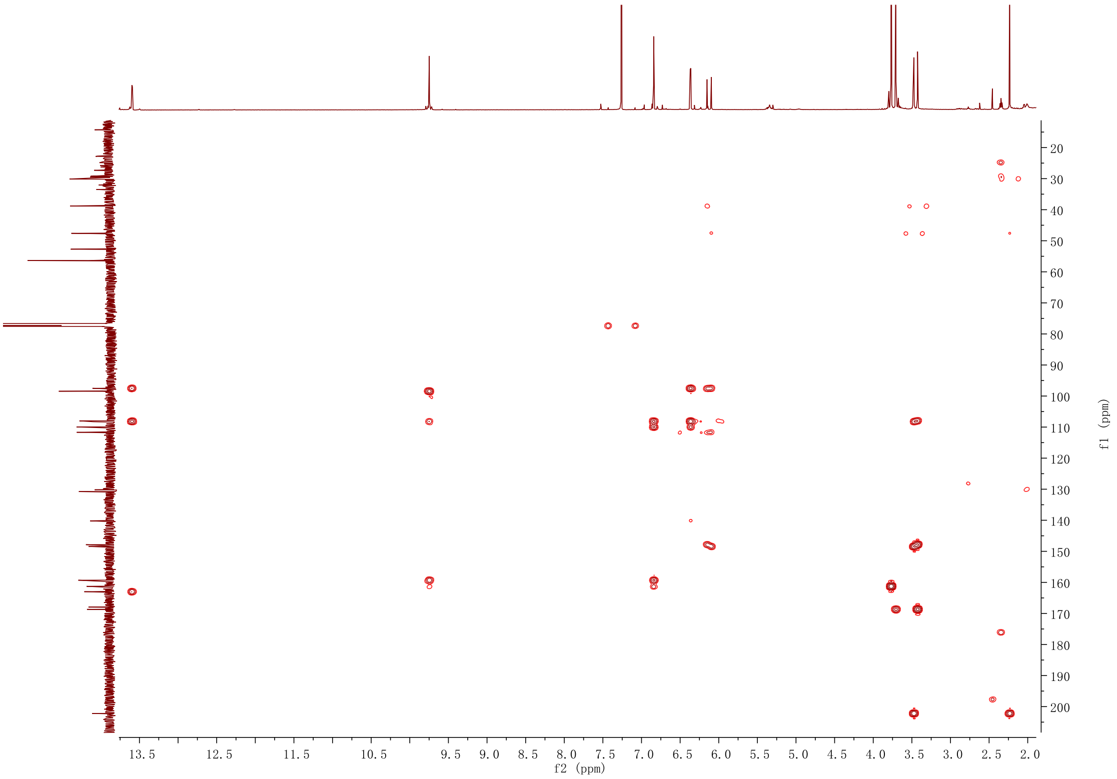

Figure S51. ^{1}H NMR spectrum (500 MHz) of 9 in chloroform-*d*

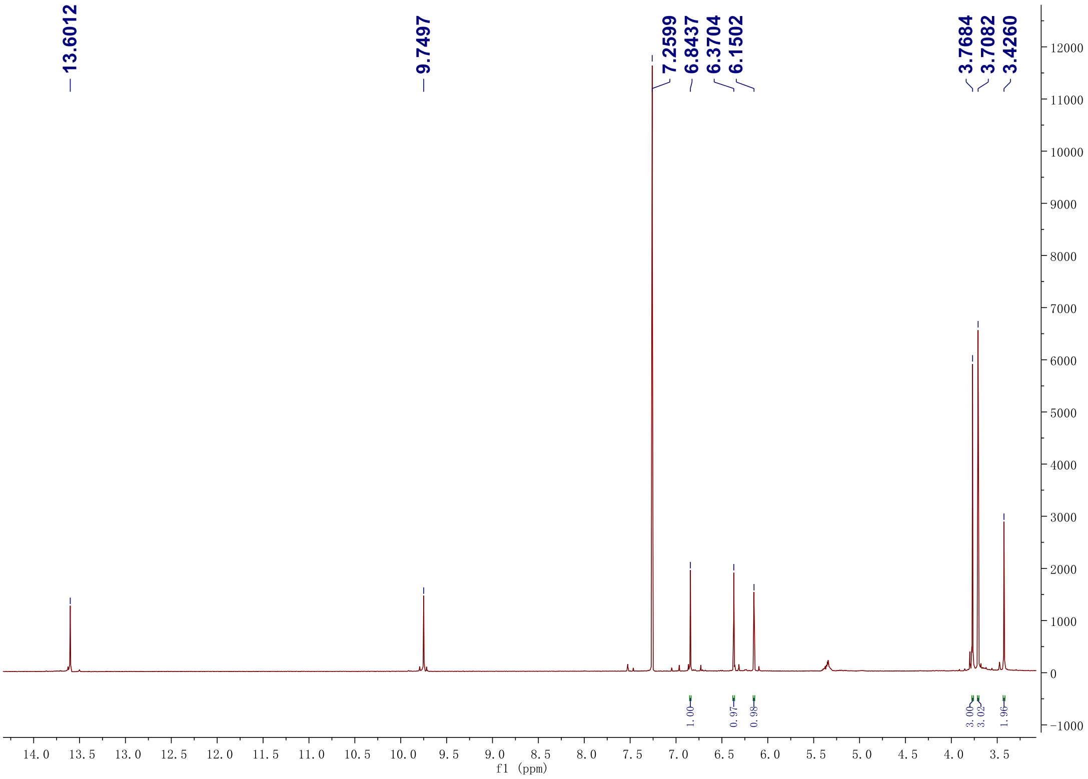

Figure S52. ^{13}C NMR spectrum (125 MHz) of 9 in chloroform-*d*

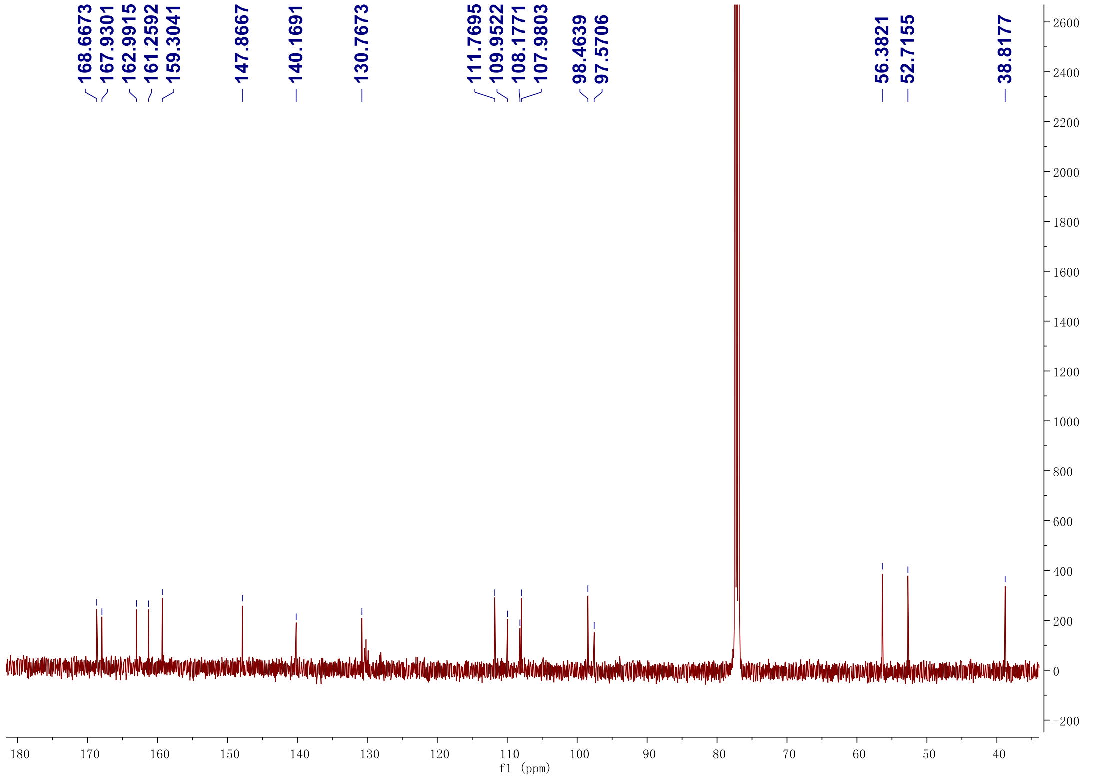

Figure S53. DEPT-135 ^{13}C NMR spectrum (125 MHz) of 9 in chloroform-*d*

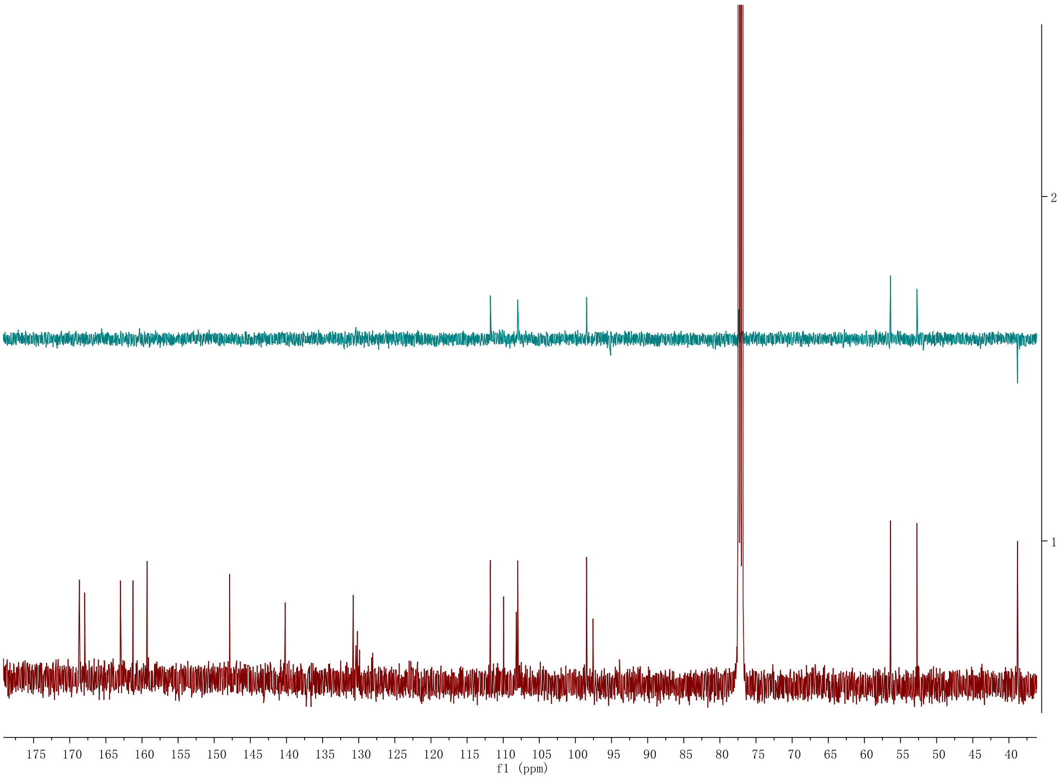

Figure S54. ^{1}H-^{1}H gCOSY NMR spectrum (500 MHz) of 9 in chloroform-*d*

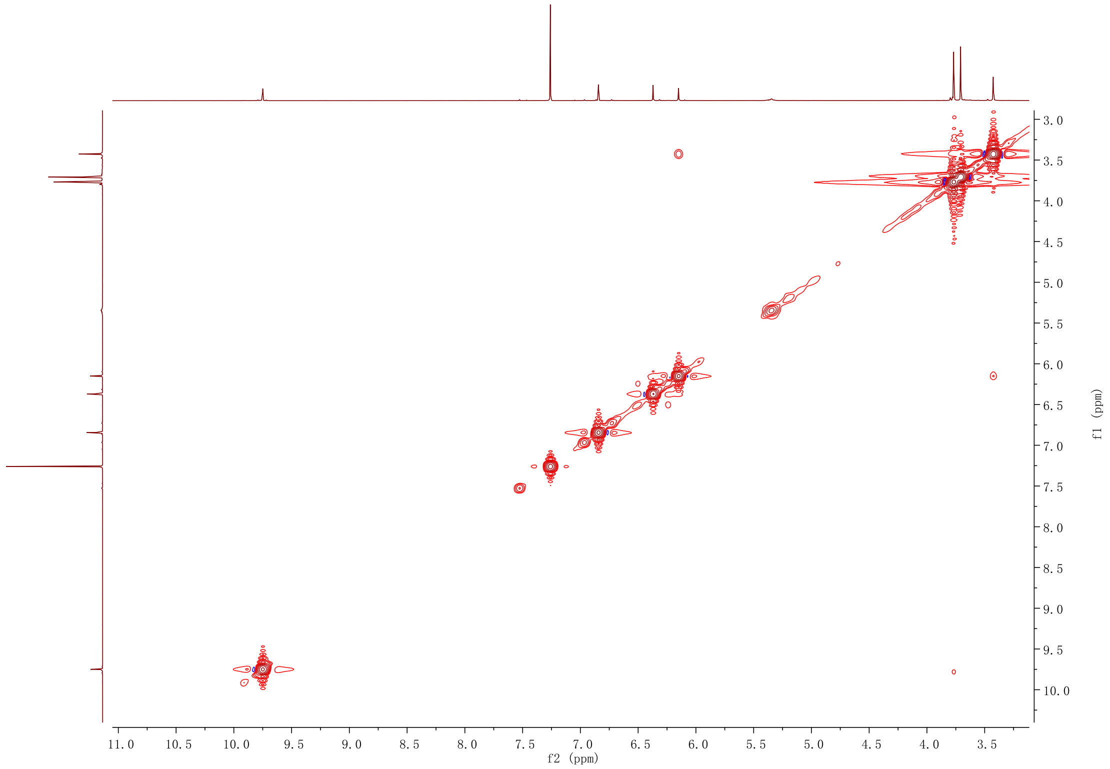

Figure S55. HSQC NMR spectrum (500 MHz) of 9 in chloroform-*d*

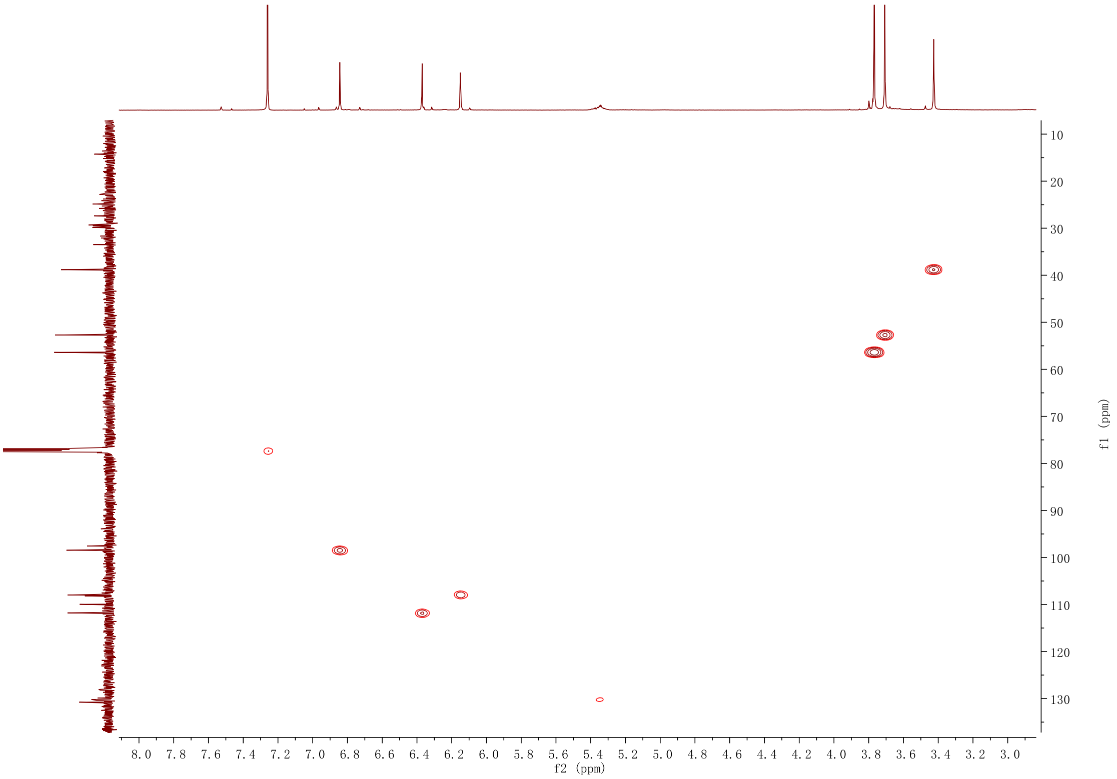

Figure S56. HMBC NMR spectrum (500 MHz) of 9 in chloroform-*d*

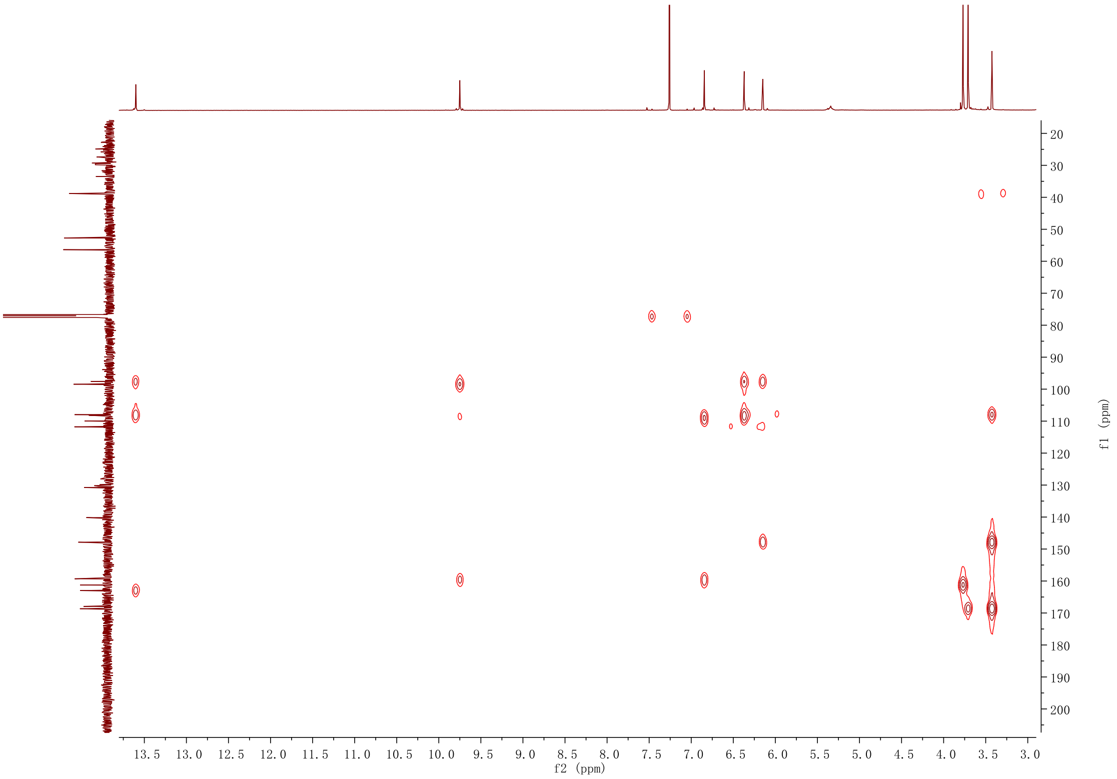

Supplement: Supplementary file 1 — Additional file 1. Supplementary data that feature Tables S1–S12 and Figures S1–S56. [file 40694_2019_72_MOESM1_ESM.pdf]
